# Integrated Bioinformatics Analysis of Serine Racemase as an Independent Prognostic Biomarker in Endometrial Cancer

**DOI:** 10.3389/fgene.2022.906291

**Published:** 2022-07-18

**Authors:** Zhiwei Cui, Jiantao Mo, Lijun Wang, Rongli Wang, Feiyan Cheng, Lihui Wang, Xinyuan Yang, Wei Wang

**Affiliations:** ^1^ Department of Obstetrics and Gynecology, The First Affiliated Hospital of Xi’an Jiaotong University, Xi’an, China; ^2^ Department of Hepatobiliary Surgery, The First Affiliated Hospital of Xi’an Jiaotong University, Xi’an, China; ^3^ Department of Anesthesiology, The First Affiliated Hospital of Xi’an Jiaotong University, Xi’an, China

**Keywords:** endometrial cancer, serine racemase, RNA modification, ferroptosis, hsa-miR-193a-5p, hsa-miR-1301-3p, TSPOAP1-AS1

## Abstract

Endometrial cancer (EC) kills about 76,000 women worldwide, with the highest incidence in industrialized countries. Because of the rise in disease mortality and new diagnoses, EC is now a top priority for women’s health. Serine racemase (SRR) is thought to play a role in the central nervous system, but its role in cancers, particularly in EC, is largely unknown. The current study starts with a pan-cancer examination of SRR’s expression and prognostic value before delving into SRR’s potential cancer-suppressing effect in patients with EC. SRR may affect the endometrial tumor immune microenvironment, according to subsequent immune-related analysis. SRR expression is also linked to several genes involved in specific pathways such as ferroptosis, N6-methyladenosine methylation, and DNA damage repair. Finally, we used the expression, correlation, and survival analyses to investigate the upstream potential regulatory non-coding RNAs of SRR. Overall, our findings highlight the prognostic significance of SRR in patients with EC, and we can formulate a reasonable hypothesis that SRR influences metabolism and obstructs key carcinogenic processes in EC.

## Introduction

With 417,000 new cases and 97,000 deaths in 2020, endometrial cancer (EC) is the sixth most common cancer in women and the seventeenth most commonly diagnosed type of cancer overall (Sung et al., 2021). There is a 10-fold difference in prevalence between regions worldwide, with Northern America and Europe ranking highest and south-central Asia ranking lowest (Sung et al., 2021). Uterine corpus cancer rates continue to rise (1.3% per year from 2007 to 2016), owing to declining fertility and increasing obesity (Miller et al., 2020). Since the mid-1970s, survival rates for all cancers other than those of the cervix and uterus have improved. EC’s most well-known risk factors are early menarche, late menopause, infertility, obesity, polycystic ovarian syndrome, and diabetes (Miller et al., 2020). According to the International Federation of Gynecology and Obstetrics, clinical staging is the most important predictor of EC (Xu et al., 2021). Patients with early-stage EC have a better prognosis than those with recurring or advanced stages, which have a poor prognosis (Giannone et al., 2019). Patients with localized disease have a 95% 5-year survival rate, while patients with distant metastasis have a 16% 5-year survival rate (Miller et al., 2020). Further research focusing on genes with higher predictive value and accelerating the transition from the molecular research phase to clinical practice is critical for improving patients’ prognoses with EC.

Serine racemase (SRR) is a pyridoxal-phosphate-dependent enzyme that converts free L-serine to D-serine. Apart from racemization, it also participates in producing pyruvate and ammonia using L-serine and D-serine as raw materials (Rani et al., 2020). SRR is found in many central nervous system tissues and peripheral tissues (Xia et al., 2004). The main product of SRR’s racemization effect is D-serine, which regulates glutamate-mediated receptor activation by interacting with the n-methyl-d-aspartate receptor’s glycine-binding site. Previous studies have extensively studied its physiological and pathological roles in the central nervous system (Balu et al., 2013; Horn et al., 2013; Ohshima et al., 2020). Unbalanced D-serine levels have been linked to Alzheimer’s disease, stroke, amyotrophic lateral sclerosis, and schizophrenia (Raboni et al., 2018). However, little is known about SRR’s role in human cancer, and its role in cancer development and tumor metabolism is unknown.

SRR’s expression and survival analysis in pan-cancer were the starting points for this research, eventually discovering its prognostic value in uterine corpus endometrial carcinoma (UCEC). SRR in UCEC was then subjected to immune-related and enrichment analyses. We also investigated the relationship between SRR and tumor mutation burden (TMB), microsatellite instability (MSI), and mutant-allele tumor heterogeneity (MATH), and the half-maximal inhibitory concentration (IC50) of commonly used chemotherapy drugs in UCEC was also investigated. Following that, we conducted clinically relevant research using univariate and multivariate analyses to determine whether SRR could be an independent predictor of the prognosis of patients with UCEC. Our study focused on SRR’s upstream regulatory non-coding RNAs (ncRNAs). We found that ncRNA-mediated downregulation of SRR in UCEC predicted negative outcomes and was linked to specific pathways such as ferroptosis, DNA damage repair, and N6-methyladenosine (m6A) methylation.

## Materials and Methods

### Gene and Protein Expression Analysis

The UCSC XENA [https://xena.ucsc.edu/, derived from The Cancer Genome Atlas (TCGA) database] was used to obtain mRNA expression data from 33 cancer tissues and corresponding types of normal tissues, 15,776 samples in total. The RNA sequencing data from TCGA and The Genotype-Tissue Expression in TPM format were processed using the Toil algorithm. These data were analyzed and compared after log2 conversion.

The RNA sequencing data and corresponding clinical information were obtained from TCGA database, totaling 11,093 samples. There were 587 samples, with 552 endometrial tumor tissues and 35 adjacent normal endometrial tissues. We obtained gene expression profiles from The Gene Expression Omnibus database (GEO) to confirm our findings: GSE17025 (including 91 UCEC samples and 12 non-tumor samples; platform, GEO: GPL570). The Human Protein Atlas (HPA) (http://www.proteinatlas.org/) website confirmed SRR expression at the mRNA and protein levels. The cell line expression matrix for 32 cancers was obtained using the Cancer Cell Line Encyclopedia database (https://portals.broadinstitute.org/ccle/about).

### Mutation, Copy Number Variation, Methylation, and Clinical-Relevant Analysis of Serine Racemase

We also obtained UCEC mutation data from TCGA database and visualized the data using the “Maftools” package (Mayakonda et al., 2018). We performed Copy Number Variation (CNV) analysis on The Gene Set Cancer Analysis website (http://bioinfo.life.hust.edu.cn/GSCA/#/). We used the “CNV” mode to get data on the CNV-related gene expression and survival analysis of SRR in UCEC. MEXPRESS (https://mexpress.be/) investigated the link between DNA methylation and SRR expression (Koch et al., 2019). MethSurv (https://biit.cs.ut.ee/methsurv/) is a web application that allows multivariate survival data based on DNA methylation to be analyzed. To prepare the region-based, methylation-related Kaplan-Meier plot, we chose the CpG site cg03846283 and split it by best (Modhukur et al., 2018). UALCAN (http://ualcan.path.uab.edu/) provides a comprehensive and complete resource for cancer-related omics data analysis (Chandrashekar et al., 2017). To collect clinically relevant data and protein expression of SRR, we used the “TCGA” and “CPTAC” modules.

### Systematic Analysis of Immune Cell Infiltration Level in Uterine Corpus Endometrial Carcinoma

We used the R package “immunedeconv,” which incorporated six cutting-edge approaches to get credible estimates of immune infiltration. We displayed the results using The Tumor Immune Estimation Resource (TIMER) algorithm. Twelve transcripts associated with immune checkpoints were identified, and their expression levels were retrieved and compared. TIMER (https://cistrome.shinyapps.io/timer/) systematically evaluated immune infiltrates in various cancer types (Li et al., 2017). The “SCNA” module was used to investigate the relationship between somatic CNV and the presence of immunological infiltrates. A two-sided Wilcoxson rank-sum test was used to compare the infiltration levels of each SCNA group to normal.

TISIDB (http://cis.hku.hk/TISIDB/index.php) is an online database that studies the interaction between tumors and the immune system by combining several heterogeneous data sources (Ru et al., 2019). Using the “subtype” module, we investigated the relationships between SRR expression, immune subtypes, and molecular subtypes in UCEC.

### Associations Between Serine Racemase Expression and TMB, MSI, MATH, and the IC50 of Four Chemotherapy Drugs in Uterine Corpus Endometrial Carcinoma

Additionally, we obtained the level4 Simple Nucleotide Variation datasets from GDC (https://portal.gdc.cancer.gov/) for all TCGA-UCEC samples processed with MuTect2 software. Using the R package Maftools, we calculated each sample’s TMB, MSI, and MATH scores. Spearman’s correlation analysis determined the relationship between SRR expression and TMB, MSI, and MATH scores. The R packages “ggradar” and “ggstatsplot” were used for visualization.

Based on the publicly available pharmacogenomics database, The Genomics of Drug Sensitivity in Cancer, we predicted each sample’s chemotherapeutic response to doxorubicin, docetaxel, cisplatin, and paclitaxel. The “pRRophetic” R package was used to implement the prediction process. Ridge regression was used to calculate the IC50. *p* < 0.05 was considered statistically significant.

### Enrichment Analysis of Serine Racemase Co-Expressed Genes

Using Spearman’s correlation, we found the top 800 genes in UCEC that were positively correlated with SRR. We converted the 800 selected genes into function annotations using the “org.Hs.eg.DB” package to identify the biological process, cellular components, molecular function, and signaling pathways that SRR may be involved in UCEC. The R package “clusterProfiler” was applied (Yu et al., 2012). Furthermore, “ggplot2” was used to visualize the results.

We used GeneMANIA software (http://www.genemania.org/) to create a functional protein-protein interaction network to identify proteins that might interact with SRR. LinkedOmics is a free website that contains multi-omics data from all 32 cancer types in TCGA (Vasaikar et al., 2018). We used Gene Set Enrichment Analysis (GSEA) in the “Linkpreter” module of LinkedOmics to perform Kyoto Encyclopedia of Genes and Genomes (KEGG) pathway analysis. The rank criterion was 0.05, and the number of simulations was 1,000.

### Predication of Upstream MicroRNAs and Long ncRNAs in Serine Racemase

To find SRR’s upstream-binding miRNAs, we used several gene interaction prediction programs, miRmap (https://mirmap.ezlab.org/), miRwalk3.0 (http://mirwalk.umm.uni-heidelberg.de/), miRDB (http://www.mirdb.org/), StarBase (http://starbase.sysu.edu.cn/), and miRactDB (https://ccsm.uth.edu/miRactDB). A miRNA was included in subsequent research if at least three different programs predicted it. These miRNAs were chosen as potential SRR-interacting candidate miRNAs.

Users could use StarBase as an encyclopedia to learn about the interactions among ncRNAs. We used StarBase to perform correlation, expression, and survival analysis of candidate miRNAs to confirm our findings. This website also predicted potential upstream lncRNAs for hsa-miR-1301-3p and hsa-miR-193a-5p and performed expression and survival analysis in UCEC. As a confirmation, we used data from The Gene Expression Profiling Interactive Analysis (GEPIA2) and the TCGA-UCEC cohort. Finally, we used the “igraph” package to create an interactive competing endogenous RNA (ceRNA) network diagram based on SRR.

We used the lncLocator database (http://www.csbio.sjtu.edu.cn/bioinf/lncLocator/) to predict the cellular localization of TSPOAP1-AS1 using its sequence, which we obtained from LNCipedia (https://lncipedia.org/).

## Results

### Expression and Prognostic Value Analysis of Serine Racemase Among 33 Cancer Types

**FIGURE 1:**
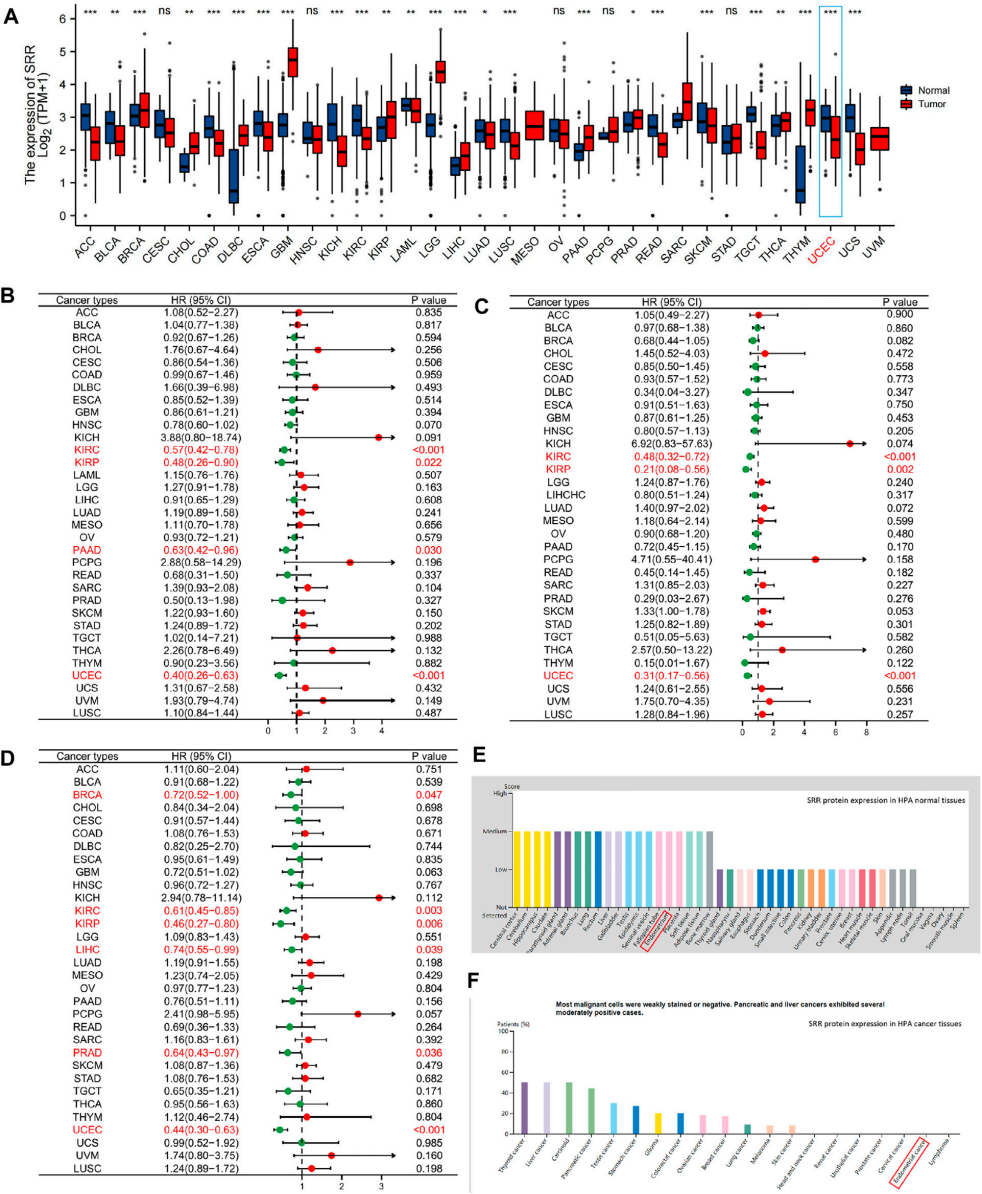
Pan-cancer expression and survival analysis of SRR. **(A)** SRR mRNA expression levels in different types of cancer and their corresponding normal tissues. **(B)** Forest plot demonstrating the relation between SRR expression and OS. **(C)** Forest plot demonstrating the relation between SRR expression and DSS. **(D)** Forest plot demonstrating the relation between SRR expression and PFI. **(E)** SRR protein expression in HPA human normal tissues. **(F)** SRR protein expression in HPA human cancer tissues. In **(A)**, * indicates *p* < 0.05, ** indicates *p* < 0.01, *** indicates *p* < 0.001, ns denotes not significantly different. In **(B–D)**, red dots represent HR > 1, green dots represent HR < 1.

The entire workflow of this study is depicted in Supplementary Figure S1. Our initial research focused on the various pan-cancer SRR expression patterns. First, we compared the expression of SRR mRNA in tumor and normal tissues. SRR mRNA was significantly lower in ACC, BLCA, COAD, ESCA, KICH, KIRC, LAML, LUAD, LUSC, READ, SKCM, TGCT, UCEC, and UCS when compared to normal controls (Figure 1A). However, it was significantly overexpressed in BRCA, CHOL, DLBC, GBM, KIRP, LGG, LIHC, PAAD, PRAD, THCA, and THYM. SRR expression was insignificant between tumor and normal tissues in only a few tumor types, including CESC, HNSC, OV, PCPG, and STAD. Different cancer cell lines had different levels of SRR expression (Supplementary Figure S2A).

We wondered if the differential expression of SRR was related to the prognosis of patients with different cancer types. As a result, we used the univariate Cox method to perform overall survival (OS), disease-specific survival (DSS), and progress-free interval (PFI) analyses on the median expression of SRR. As shown in Figure 1B, SRR expression significantly increased the OS of patients in KIRC [hazard ratio (HR) = 0.57, *p* < 0.001], KIRP (HR = 0.48, *p* = 0.022), PAAD (HR = 0.63, *p* = 0.030), and UCEC (HR = 0.40, *p* < 0.001). The relationship between SRR expression and DSS is shown in Figure 1C. High SRR expression was found to be a protective factor in KIRC (HR = 0.48, *p* < 0.001), KIRP (HR = 0.21, *p* = 0.002), and UCEC (HR = 0.31, *p* < 0.001). In six cancer types, high SRR expression significantly improved PFI, as shown in Figure 1D. BRCA (HR = 0.72, *p* = 0.047), KIRC (HR = 0.61, *p* = 0.003), KIRP (HR = 0.46, *p* = 0.006), LIHC (HR = 0.74, *p* = 0.039), PRAD (HR = 0.64, *p* = 0.036), and UCEC (HR = 0.44, *p* < 0.001). SRR may function as a tumor suppressor gene in some cancers, such as KIRC and UCEC, based on expression and survival analysis.

### Serine Racemase is Downregulated in Uterine Corpus Endometrial Carcinoma, While Its Upregulation Predicts Favorable Outcomes

**FIGURE 2:**
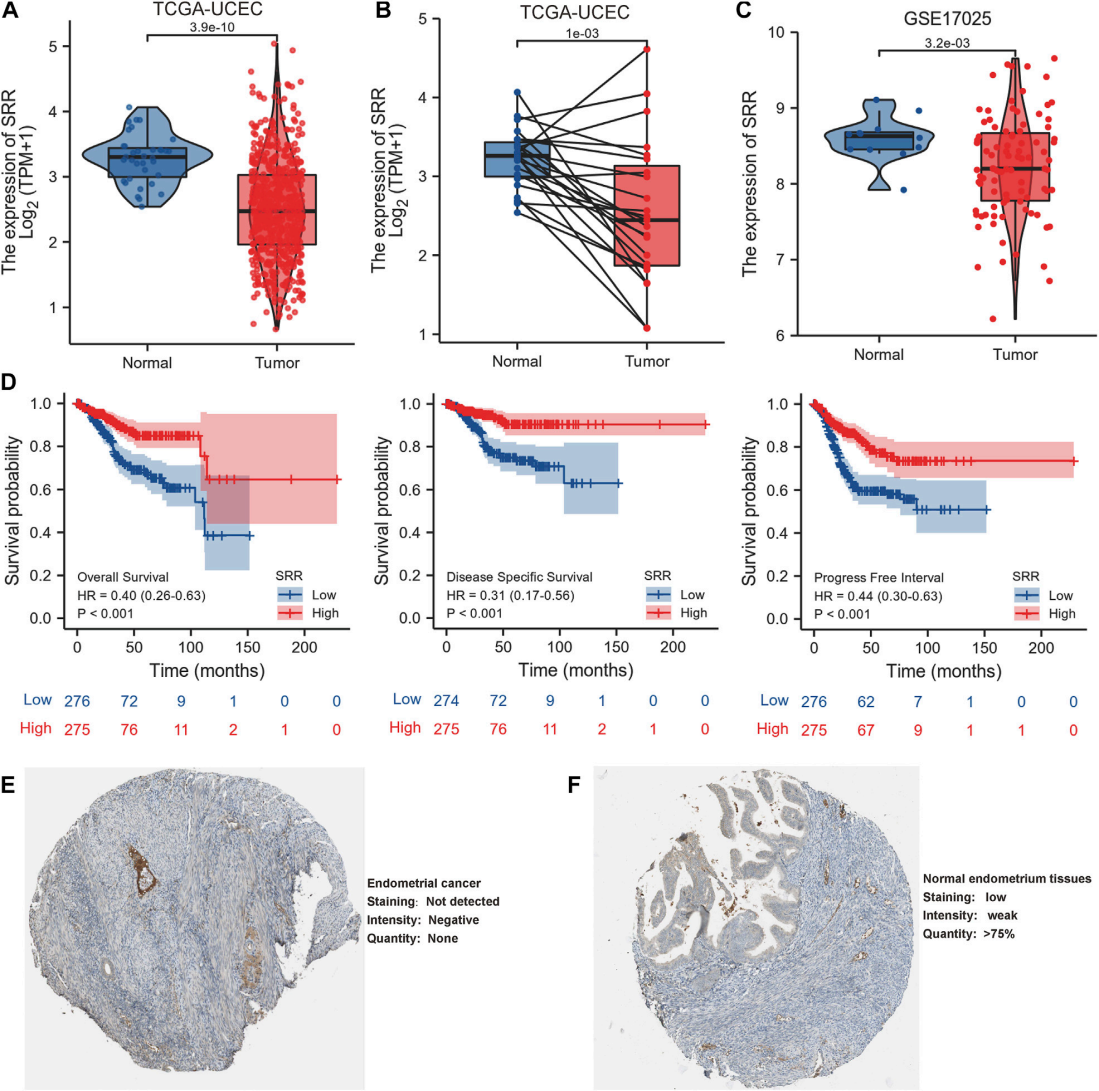
Expression and prognostic value of SRR in UCEC. **(A)** SRR mRNA expression level in UCEC tissues (*n* = 552) compared with normal tissues (*n* = 35). **(B)** SRR mRNA expression is lower in UCEC tissues than in paired adjacent normal tissues (*n* = 23). **(C)** Validation of SRR expression by analyzing data from GSE17025. **(D)** OS, DSS, and PFI survival Kaplan-Meier curves of SRR in TCGA - UCEC patients. **(E,F)** Validation of SRR at the translational level using HPA database (immunohistochemistry).

In comparison to normal tissues, we used the HPA database to confirm the mRNA and protein expression levels of SRR in UCEC. We discovered that SRR expression was low at the mRNA (Supplementary Figures S2B,C) and protein levels (Figures 1E,F). In the UALCAN CPTAC samples, the difference in protein expression was confirmed (Supplementary Figure S3G). We then looked at SRR expression in 552 UCEC tissues and 35 adjacent normal tissues using the TCGA-UCEC cohort. SRR expression was significantly low in UCEC (*p* = 3.9e-10), consistent with previous findings (Figure 2A). The difference in SRR expression in 23 paired tumors and tumor-adjacent normal tissues supported our findings (Figure 2B). As external validation, we used GSE17025 datasets, which included 12 normal and 91 tumor tissues (Figure 2C). SRR expression was downregulated in UCEC using standard IHC labeling collected from HPA (Figures 2E,F).

According to Kaplan-Meier survival curves, patients with higher SRR expression had better OS, DSS, and PFI (Figure 2D). SRR’s prognostic value in UCEC was also confirmed using the Kaplan-Meier Plotter database. Following the observation of 2, 5, and 10-year OS and relapse-free survival, we discovered that increased SRR expression was favorable (Supplementary Figures S3A–F).

### Relation Between Serine Racemase and Immunity in Uterine Corpus Endometrial Carcinoma

**FIGURE 3:**
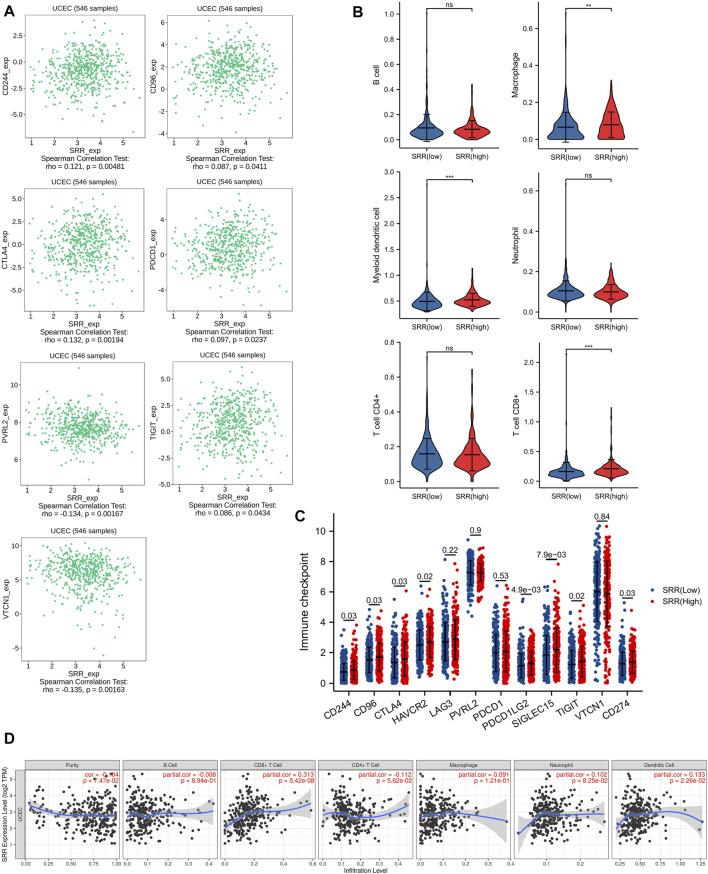
Immune-related analysis of SRR in UCEC. **(A)** Seven immune inhibitors significantly correlated with SRR expression using TISIDB. **(B)** Violin plots showing the different immune infiltration levels in SRR high and low groups. **(C)** The comparison of the expression of immune checkpoint-related genes between UCEC SRR-high expression group (red) and SRR-low expression group (blue); the number indicates the *p*-value. **(D)** Correlation between SRR expression and immune cell infiltration levels in UCEC.

We used the TIMER algorithm to determine the percentage of six different types of immune cells in the UCEC microenvironment to investigate the possible involvement of SRR in the UCEC immune microenvironment. The Wilcoxson rank-sum test revealed that myeloid dendritic cell, T cell CD8^+^, and macrophage infiltration levels were higher in the SRR high-expression group than in the SRR low-expression group (Figure 3B). Then, as shown in Figure 3D, we used the TIMER database to confirm that in UCEC, SRR expression was not significantly linked with tumor purity (R = −0.104, *p* = 7.47e−02), but it was remarkably and positively correlated with CD8^+^ T cell infiltration (*r* = 0.313, *p* = 5.42e−08) and dendritic cell infiltration (*r* = 0.133, *p* = 2.26e−02). Moreover, in patients with UCEC, there was an overall positive correlation between immune cell infiltration and cumulative survival (Supplementary Figure S4A).

As shown in Supplementary Figure S4B, deletion of SRR at the chromosome arm level significantly reduced CD8^+^ T cell infiltration (*p* < 0.001), macrophage infiltration (*p* = 0.006), and dendritic cell infiltration (*p* < 0.001). Moreover, we discovered that SRR expression differed between immune and molecular subtypes. SRR expression was lowest in the C4 (lymphocyte depleted) immune subtype and highest in the C3 (inflammatory) immune subtype, with patients in the C3 subtype having better UCEC prognoses (Supplementary Figure S4D) (Thorsson et al., 2018). SRR was also higher in the MSI and POLE molecular subtypes, and patients with UCEC in these two subtypes had better prognoses (Supplementary Figure S4E) (Urick and Bell, 2019).

CD244, CD96, CTLA4, HAVCR2, LAG3, PVRL2, PDCD1, PDCD1LG2, SIGLEC15, TIGIT, VTCN1, and CD274 were chosen as immune-checkpoint transcripts, and the expression differences of these 12 genes were compared between normal and patients with UCEC, as well as between UCEC SRR-high and SRR-low expression groups. CTLA4, HAVCR2, PVRL2, PDCD1, SIGLEC15, TIGIT, and VTCN1 expression levels were higher in tumor tissues, while CD244, LAG3, PDCD1LG2, and CD274 expression levels were found to be lower (Supplementary Figure S4C). Additionally, patients with high levels of SRR expression had significantly higher levels of CD244, CD96, CTLA4, HAVCR2, PDCD1LG2, SIGLEC15, TIGIT, and CD274 expression (Figure 3C). The TISIDB database was used to investigate Spearman’s correlations between SRR expression and immunoinhibitors. SRR expression was significantly correlated with a total of seven immunoinhibitors, five of which were positively correlated with SRR, including CD244 (rho = 0.12, *p* = 4.81e−03), CD96 (rho = 0.09, *p* = 4.11e−02), CTLA4 (rho = 0.13, *p* = 1.94e−03), PDCD1 (rho = 0.10, *p* = 2.37e−02), and TIGIT (rho = 0.09, *p* = 4.34e−02), while the remaining two were negatively correlated with SRR, including PVRL2 (rho = −0.13, *p* = 1.67e−03) and VTCN1 (rho = −0.14, *p* = 1.63e−03) (Figure 3A).

### Associations of TMB, MSI, MATH, and Chemotherapeutic Drug Sensitivity With Serine Racemase Expression in Uterine Corpus Endometrial Carcinoma

**FIGURE 4:**
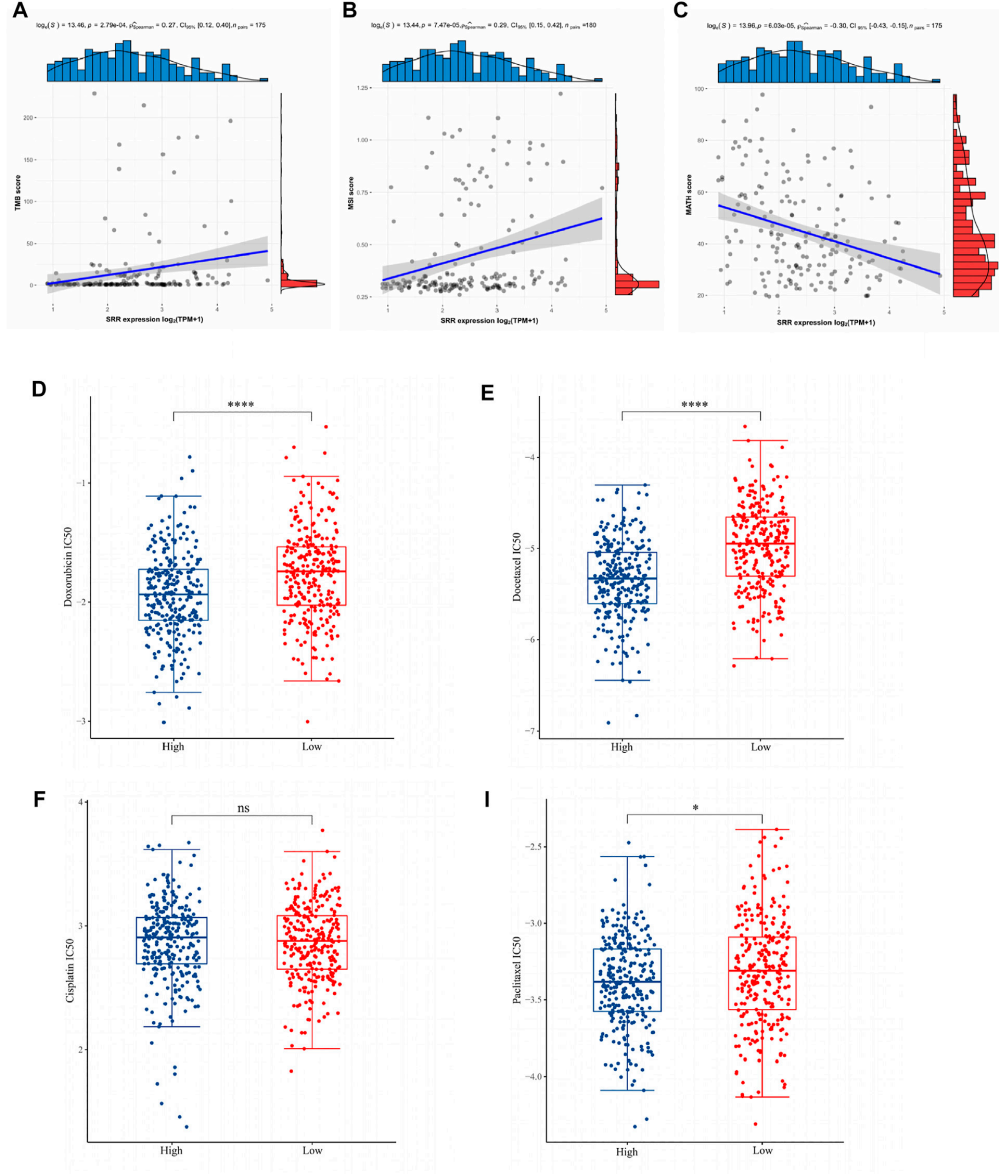
Associations of TMB, MSI, MATH, and chemotherapeutic drug sensitivity with SRR expression in UCEC. **(A–C)** Scatterplots display the Spearman correlation between SRR expression and TMB **(A)**, MSI **(B)**, and MATH **(C)** scores in UCEC. The abscissa represents the expression distribution of SRR gene expression, and the ordinate is the expression distribution of the TMB/MSI/MATH scores. The density curve on the right represents the TMB/MSI/MATH score, and the upper-density curve represents the SRR gene expression distribution trend. **(D–I)** Chemotherapy drug sensitivity analysis. The blue and red color represent the UCEC SRR-high expression and SRR-low expression group, respectively. The ordinate represents the distribution of the IC50 score of doxorubicin **(D)**, docetaxel **(E)**, cisplatin **(F)**, and paclitaxel **(I)**. *p < 0.05, ****p < 0.001, ns denotes not significantly different.

TMB could be used as a biomarker to evaluate the efficacy of immunotherapy in the treatment of various cancers. MSI has also been proposed as a cancer immunotherapy prognostic biomarker. MATH is a method for calculating the genetic heterogeneity of a tumor. Then, as shown in Figures 4A–C, the associations between TMB, MSI, and MATH scores and SRR expression of each sample in UCEC were evaluated. In UCEC, there was a significant correlation between SRR expression and TMB (*r* = 0.27, *p* = 2.79e−04). In UCEC, SRR was positively correlated with MSI (*r* = 0.29, *p* = 7.47e-05). The coefficient r of Spearman’s correlation between SRR and MATH was −0.30, with a *p*-value of 6.03e−05. These findings suggested that SRR could be a promising target for immune therapy in UCEC.

We selected four commonly used chemotherapeutic agents for UCEC based on previously published authoritative literature (Brooks et al., 2019; Nomura et al., 2019). The IC50 of three drugs, doxorubicin (Figure 4D), docetaxel (Figure 4E), and paclitaxel (Figure 4I), was found to be significantly higher in the SRR-low expression group, implying that patients with SRR-high expression were more sensitive to these three drugs. In contrast, there was no significant difference in the IC50 of cisplatin between the two groups (Figure 4F).

### Serine Racemase Co-Expressed Genes Subjected to Gene Ontology, KEGG, and GSEA in Patients With TCGA-UCEC

**FIGURE 5:**
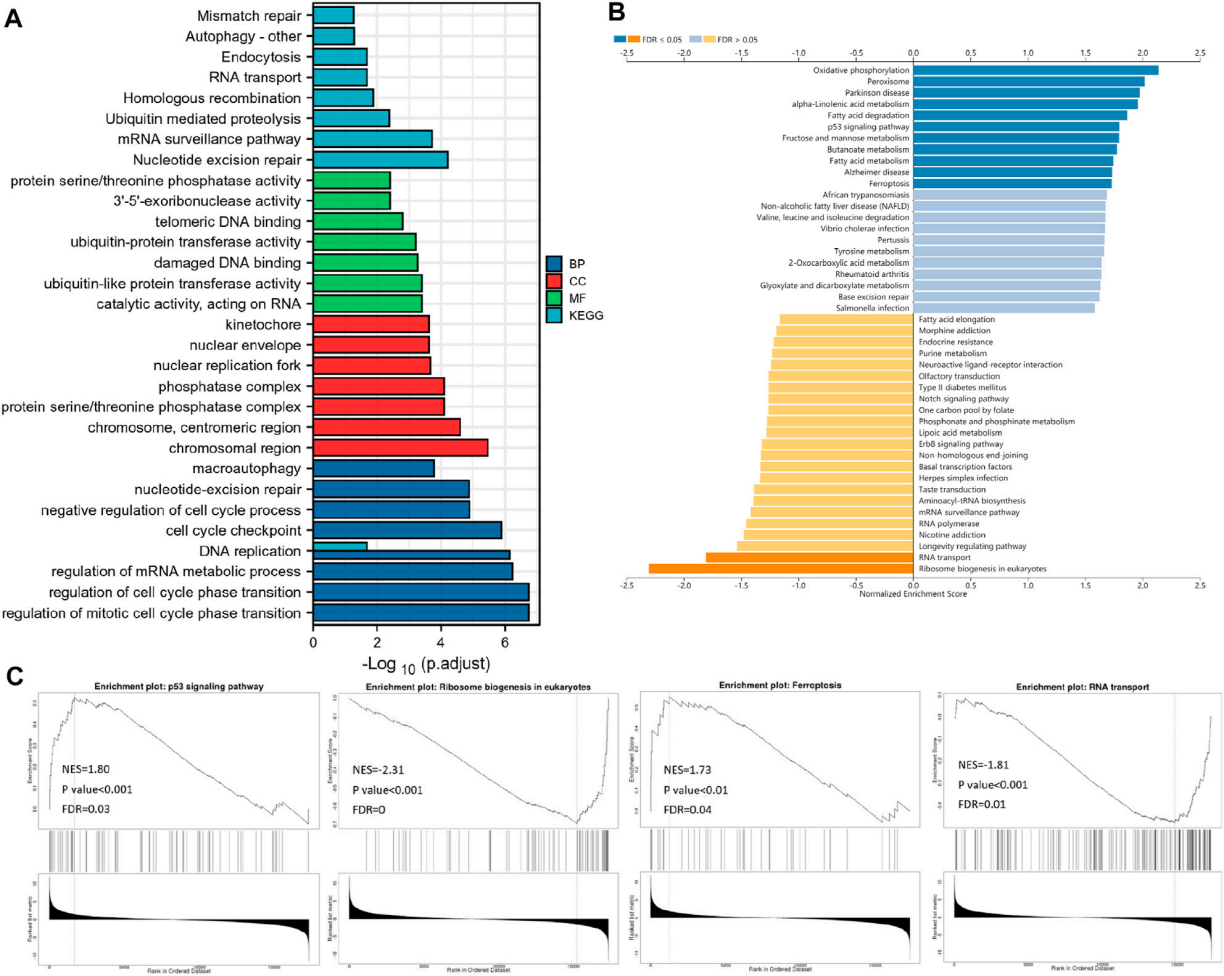
Functional enrichment analysis. **(A)** Bar plot displays the GO and KEGG analysis of the top 800 genes, which show the most positive correlation with SRR by data from TCGA. LinkedOmics-based gene set enrichment analyses (GSEA) of SRR-associated pathways are shown in **(B)** and **(C)**.

A correlation analysis was used to predict the likely activities and linked pathways of SRR in UCEC. The top 50 genes that positively and negatively correlated with SRR in UCEC are displayed in Supplementary Figures S5B,C. The top 800 genes with strong and positive correlations with SRR were then analyzed for GO and KEGG enrichment. According to GO analysis, SRR was primarily involved in cell replication and DNA damage repair processes such as DNA replication, cell cycle regulation, nucleotide mismatch repair, chromosome structure, and 3′-5′-exoribonuclease activity. Additionally, SRR’s involvement in the ubiquitination process was likely to affect ubiquitin-protein and ubiquitin-like protein transferase activity. Furthermore, SRR was strongly linked to several DNA and RNA-related pathways, including nucleotide excision repair, the mRNA surveillance system, homologous recombination, DNA replication, RNA transport, and mismatch repair (Figure 5A).

According to the GSEA KEGG analysis, SRR co-expressed genes were involved in oxidative phosphorylation, peroxisome, Parkinson’s disease, alpha-linolenic acid metabolism, fatty acid degradation, fructose and mannose metabolism, p53 signaling pathway, butanoate metabolism, Alzheimer’s disease, fatty acid metabolism, and ferroptosis. However, in eukaryotes, RNA transport and ribosome biogenesis were inhibited (Figure 5B). Four interesting pathways were selected and were displayed in Figure 5C.

We used GeneMANIA software to predict and visualize the interaction network of SRR’s potential interactive proteins. Twenty SRR-interacting proteins were discovered, and they were found to interact closely with SDS, SDSL, FBXO22, POLR1C, LARS1, IARS1, PRELID1, THNSL2, CBSL, CBS, and DHRS11 (Supplementary Figure S5A).

### Correlation Between SRR and RNA Methylation Modification-Related Genes, Ferroptosis-Related Genes, Mismatch Repair-Related Genes, and Tumor Suppressor Genes in UCEC

The SRR gene was primarily involved in DNA damage repair, ferroptosis, ubiquitination, and RNA-related pathways. As a result, we used a comprehensive and detailed analysis to understand SRR better.

**FIGURE 6:**
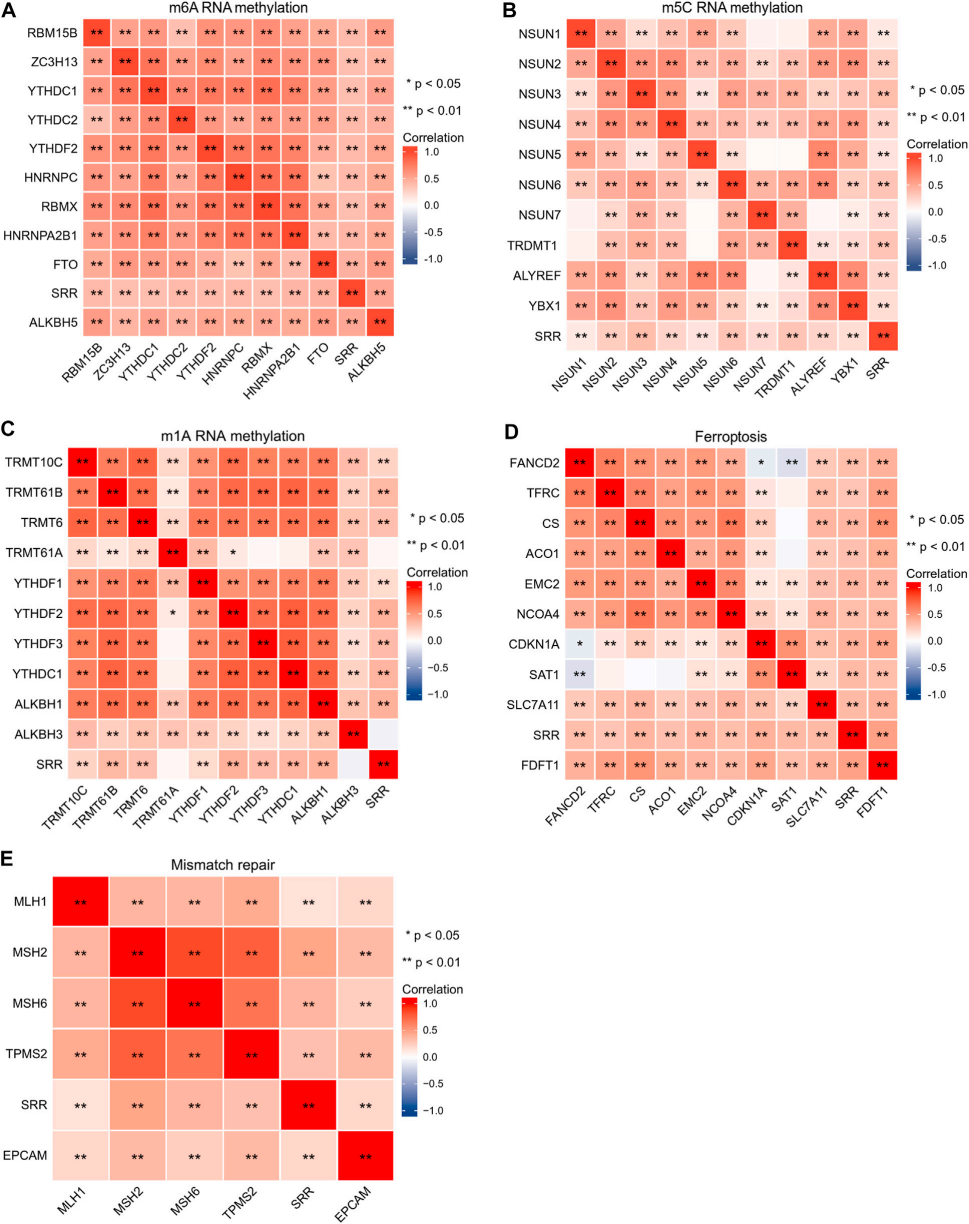
Correlation analysis of SRR with m6A RNA methylation-related genes **(A)**, m5C RNA methylation-related genes **(B)**, m1A RNA methylation-related genes **(C)**, ferroptosis-related genes **(D)**, and mismatch repair-related genes **(E)** in UCEC. Red shows positive correlation, and blue shows negative correlation. The stronger the correlation, the darker the color. *p < 0.05, **p < 0.01.

The importance of RNA methylation in the occurrence and progression of cancer has long been recognized, and there have been over 170 different RNA chemical alterations discovered to date, with m6A, N1-methyladenosine (m1A), and 5-methylcytosine (m5C) being the most well-studied (Esteve-Puig et al., 2020). From previous studies, Figures 6A–C compile the correlations among SRR and RNA methylation modification-related genes (Li et al., 2019; Du et al., 2020; Li et al., 2021). According to the correlation heatmap, the m6A-methylation-related genes covering writers (RBM15B, ZC3H13, and RBMX), readers (YTHDC1, YTHDC2, YTHDF2, HNRNPC, and HNRNPA2B1), and erasers (FTO and ALKBH5) showed significant and positive correlations with one another. SRR showed significant correlations with the 10 m6A-genes in UCEC (Figure 6A). SRR had positive and significant correlations with 10 m5C-genes, as shown in Figure 6B. SRR had significant associations with all m1A-genes except TRMT61A and ALKBH3, as shown in Figure 6C.

Ferroptosis, a unique mode of cell death, is linked to cancer initiation, progression, and suppression (Wang Y et al., 2020). Mismatch repair genes are involved in suppressing cancer-causing mutations and the induction of protective mechanisms in response to the challenge of irreversible DNA damage (Ijsselsteijn et al., 2020). Genes associated with ferroptosis and genes involved in mismatch repair were selected from previous studies (Deshpande et al., 2020; Liu Z et al., 2020). FANCD2 was negatively correlated with SAT1 and CDKN1A in SRR and ferroptosis-associated genes, while SAT1 had no significant correlation with TFRC, CS, and ACO1, and the remaining correlations were all significant and positive (Figure 6D). As shown in Figure 6E, SRR and five mismatch repair genes are closely and positively correlated.

Moreover, we included over 200 DNA damage repair genes (Jinjia et al., 2019). We prepared a ring heat map (Supplementary Figure S6A) after calculating their correlation with SRR in UCEC, indicating that SRR is likely involved in the DNA damage repair process. In UCEC, TIMER2.0 was used to investigate the relationship between SRR and 30 common tumor suppressor genes (Supplementary Figure S6B). The significant and positive correlations suggested that SRR, like many other tumor suppressor genes, may work together to fight cancer, especially in UCEC.

Finally, we divided patients with TCGA-UCEC into two groups based on median SRR expression. We included more ferroptosis and m6A methylation-related genes in the expression comparison between the two groups. In most of these genes, we found significant and differential expression between the two groups (Supplementary Figures S7A,B).

### Serine Racemase Mutation, Copy Number Variation, and Methylation Analysis

**FIGURE 7:**
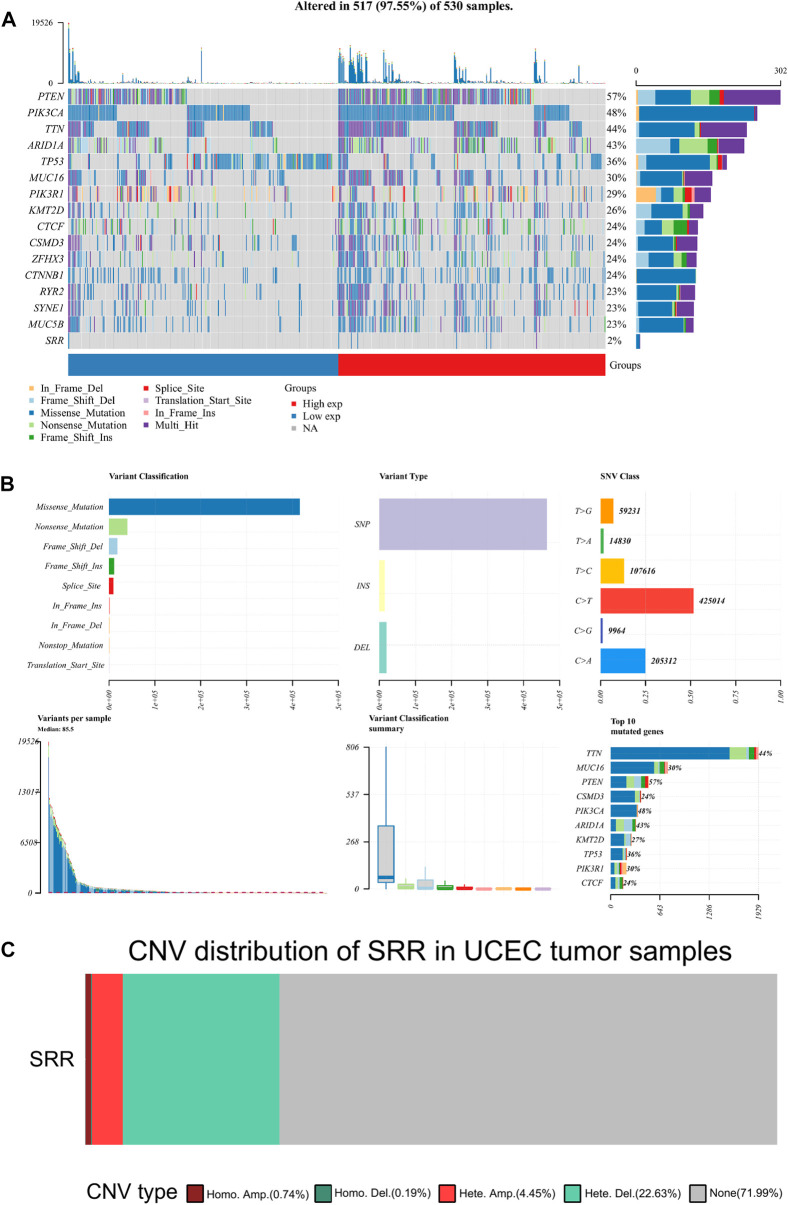
Mutation and copy number variation of SRR in UCEC. **(A)** Oncoplot displaying the somatic landscape of the UCEC cohort. Genes are ordered by their mutation frequency, and samples are ordered according to SRR expression indicated by the annotation bar (bottom). The waterfall plot shows the mutation information of each gene within each sample. **(B)** Cohort summary plot displaying the distribution of variants according to variant classification, type, and SNV class. The lower part depicts mutation load for each sample and variant classification type. A stacked bar plot shows the top 10 mutated genes. **(C)** The distribution of SRR CNV type in UCEC patients. Different colors represent different CNV types.

The mutation data were visualized and analyzed using the R package Maftools. PTEN, PIK3CA, TTN, ARID1A, and TP53 were the top five genes with the highest mutation rate in UCEC. In all UCEC samples, the SRR mutation rate was 2% (Figure 7A). Missense mutation and single nucleotide polymorphism were the most common variant classifications and variant types. The top single nucleotide variant class was C > T (Figure 7B). The CNV alteration frequency of SRR in UCEC was approximately 28%, the vast majority of which were heterozygous deletions and amplifications (Figure 7C). The bubbles represent the percentage of heterozygous and homozygous CNV in Supplementary Figures S8A,B. SRR CNV in UCEC was positively correlated with mRNA RESM, with a Spearman correlation of 0.5, false discovery rate (FDR) < 0.0001 (Supplementary Figure S8C). In UCEC, the survival difference between CNV and wild type groups is summarized in Supplementary Figure S8D. The CNV and wild-type groups had significant log-rank *p*-values for all prognosis-related parameters, including OS, DSS, disease-free interval (DFI), and progression-free survival (PFS) (Supplementary Figure S8D).

Patients with UCEC were divided into two groups based on SRR median expression: high and low. A bar chart was plotted to depict the mutation frequency difference between the two groups for the top five mutated genes. PTEN, PIK3CA, TTN, and ARID1A mutation frequencies were higher in the SRR-high expression group, and the results were statistically significant. However, in the SRR-high expression group, the TP53 gene was less frequently mutated (Supplementary Figure S9A).

We hypothesized that DNA methylation was responsible for SRR’s downregulation. The relationship between SRR expression and its promoter methylation level was then determined using MEXPRESS. Four CpG islands were significantly associated with SRR expression: cg02945294, cg22556056, cg21745320, and cg03846283. The first two had significant and negative associations with SRR expression (Supplementary Figure S9B). According to Methsurv online tool, patients with higher methylation levels in the promoter region of cg02945294 had a poor prognosis, with an HR = 3.113 and a likelihood ratio (LR) test *p*-value = 1e−04 (Supplementary Figure S9C). This result supported our previous conclusion that high SRR expression predicted a better prognosis in patients with UCEC.

### Association Between Serine Racemase Expression and Clinicopathological Variables

**FIGURE 8:**
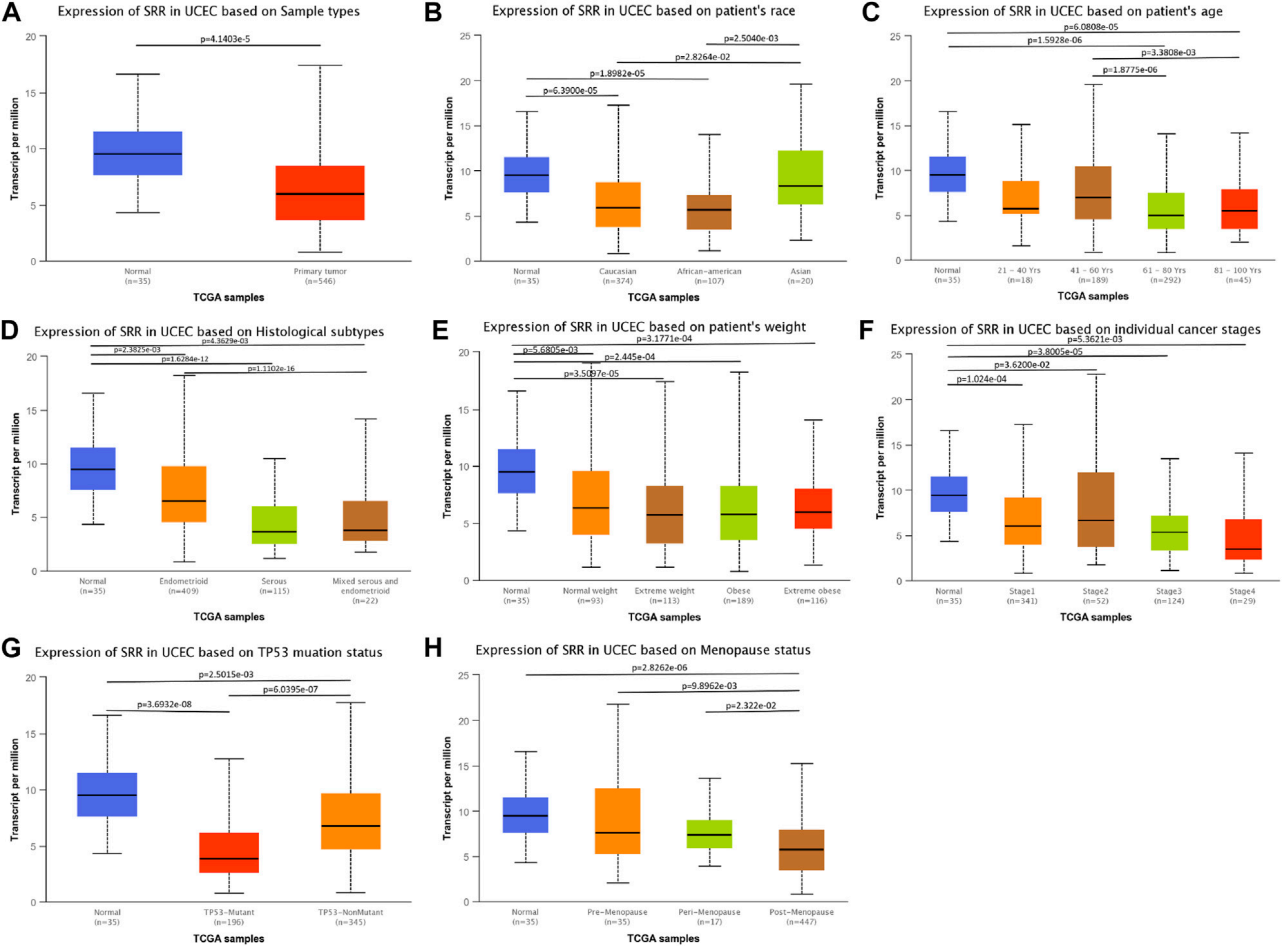
Associations between SRR expression and different clinicopathological variables including sample types **(A)**, patient’s race **(B)**, patient’s age **(C)**, histological subtypes **(D)**, patient’s weight **(E)**, cancer stages **(F)**, TP53 mutation status **(G)**, and menopause status **(H)** in TCGA UCEC patients using the UALCAN datasets.

In UCEC, we found a link between SRR expression and clinical characteristics. Age (*p* < 0.001), histological type (*p* < 0.001), histologic grade (*p* = 0.044), menopause status (*p* = 0.002), and residual tumor (*p* = 0.014) were all found to be significantly related to SRR. Additionally, SRR was only marginally related to the clinical stage (*p* = 0.067) (Supplementary Table S1). Furthermore, patients of normal tissues (Figure 8A), Asian race (Figure 8B), age <60 years (Figure 8C), histological type of endometrioid (Figure 8D), normal weight (Figure 8E), earlier clinical staging (Figure 8F), TP53-nonmutant status (Figure 8G), and pre-menopause (Figure 8H) were found to have higher levels of SRR. SRR expression was also negatively and weakly correlated with the clinical stage (Spearman’s *r* = −0.1, *p* = 2.52e−02) (Supplementary Figure S10B) and grading (Spearman’s *r* = −0.164, *p* = 1.46e−04) (Supplementary Figure S10D) in the TISIDB database, indicating that SRR expression decreased as clinical stage and grading increased.

Logistic regression analysis was used to confirm the relationship between SRR expression and clinicopathological variables using the SRR high-low dichotomy. High SRR expression was found to be significantly and positively correlated with stage I/II [odds ratio (OR) = 1.617, *p* = 0.011], G1/2 (OR = 1.540, *p* = 0.014), histological type of endometrioid (OR = 4.448, *p* < 0.001), age ≤ 60 (OR = 2.241, *p* < 0.001), R0 (OR = 2.938, *p* = 0.005), and pre- and peri-menopause status (OR = 2.766, *p* = 0.001) (Supplementary Table S2). As a result, the results of logistic regression were very similar to what we had previously discussed.

### Additional Investigation of the Clinical and Prognostic Significance of Serine Racemase in Patients With Uterine Corpus Endometrial Carcinoma

**FIGURE 9:**
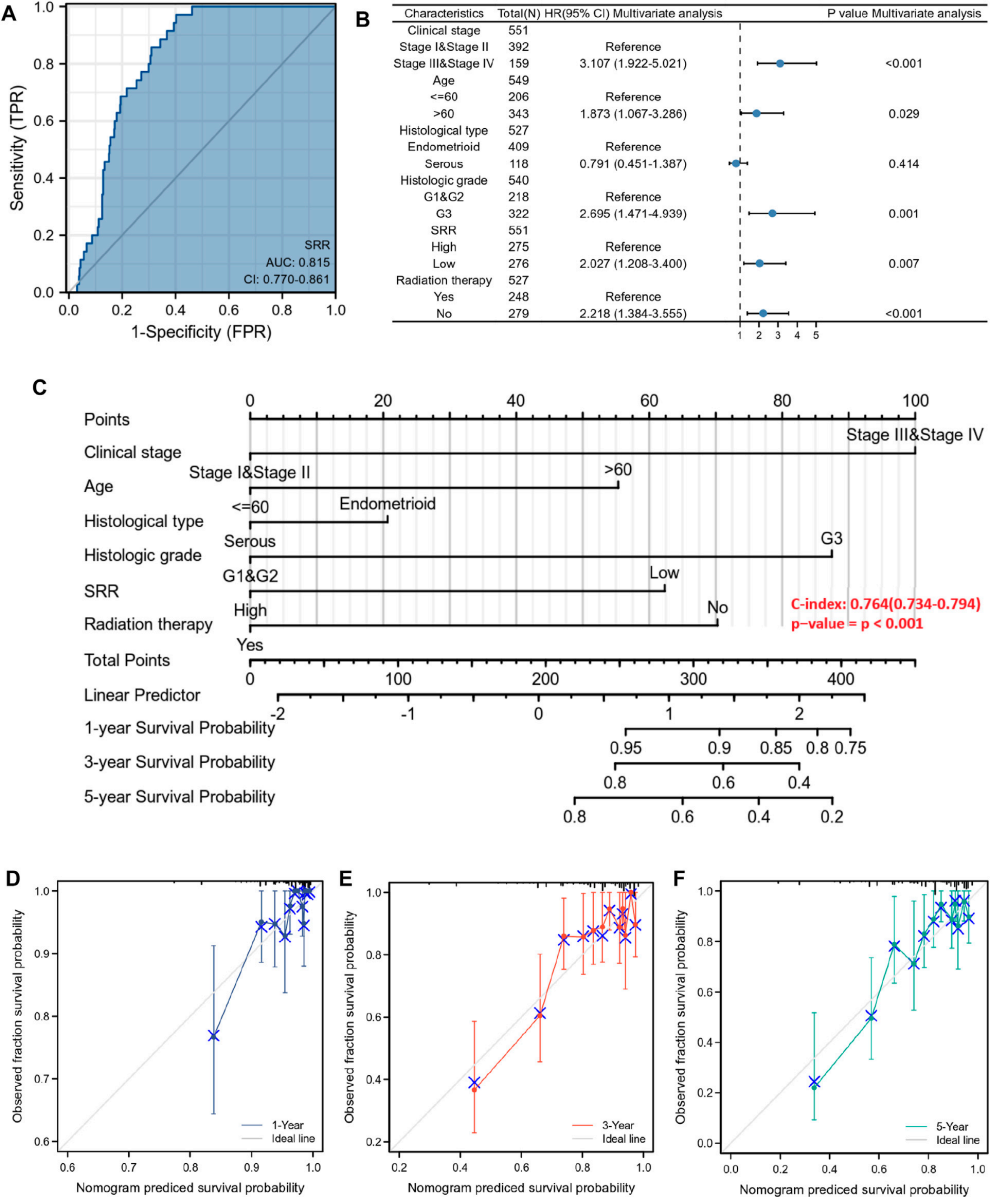
Diagnostic and prognostic value of SRR in UCEC patients. **(A)** ROC analysis of SRR shows good discrimination power between tumor and normal tissues. **(B)** Forest plot shows the results of the multivariate Cox regression analysis of the clinicopathological characteristics affecting the OS of UCEC patients. **(C)** A nomogram for predicting the 1-, 3-, and 5-year OS probability for UCEC patients. **(D–F)** Calibration curves of 1-, 3-, and 5-year OS of UCEC patients. The ordinate represents the actual OS, while the abscissa represents the nomogram-predicted OS.

First, we used a ROC curve to assess the sensitivity and specificity of the SRR gene in predicting its diagnostic value of UCEC. SRR’s area under the curve was 0.815, indicating significant predictive power in predicting UCEC and normal (Figure 9A). A univariate Cox proportional hazards regression analysis assessed the factors influencing patients’ OS. Higher clinical stage (III/IV) (HR = 3.543, *p* < 0.001), age >60 years (HR = 1.847, *p* = 0.01), serous type of histology (HR = 2.646, *p* < 0.001), higher histologic grade (G3) (HR = 3.281, *p* < 0.001), lower SRR expression (HR = 2.494, *p* < 0.001), and without radiation therapy (HR = 1.684, *p* = 0.018) were among the clinicopathological factors linked to shorter OS (Supplementary Table S3). Following that, we performed a multivariate Cox regression analysis and discovered that lower SRR expression was still an independent risk factor (HR = 2.027, *p* = 0.007), along with clinical stage (HR = 3.107, *p* < 0.001), age (HR = 1.873, *p* = 0.029), histologic grade (HR = 2.695, *p* = 0.001), and radiation therapy (HR = 2.218, *p* < 0.001) (Supplementary Table S3; Figure 9B). Both univariate and multivariate analyses were performed at the DSS and PFI levels. Clinical stage, histological grade, SRR expression, and radiation therapy were independent prognostic factors for DSS (Supplementary Figure S11C; Supplementary Table S4). Clinical stage, SRR expression, and surgical approach were independent prognostic factors for PFI (Supplementary Figure S11D; Supplementary Table S5).

Finally, a prognostic nomogram for 1-, 3-, and 5-year OS patients with UCEC was created using the previously described results from multivariate Cox regression analysis. A point scale was used to assign points to these variables, and the sum of the points assigned to each variable was rescaled to a range of 0–100 using multivariate analysis. By adding the points from each variable, the total points were calculated. The nomogram model had a C-index of 0.764 (95% confidence interval: 0.734–0.794, *p* < 0.001) (Figure 9C). The 1-, 3-, and 5-year calibration curves were close to the ideal line, indicating that the predicted and observed values were aligned (Figures 9D–F). All patients were divided into low- and high-risk score groups based on the median value of the nomogram model’s risk score. Kaplan-Meier analysis revealed that patients with a high-risk score had a worse prognosis (HR = 3.52, *p* < 0.001) (Supplementary Figure S11B). The distribution of the risk score and the survival status of patients with UCEC is shown in Supplementary Figure S11A. As the risk score increased, it was observed that patients’ survival time decreased and their risk of death increased.

### Prediction of Upstream MiRNAs That May Interact With Serine Racemase

**FIGURE 10:**
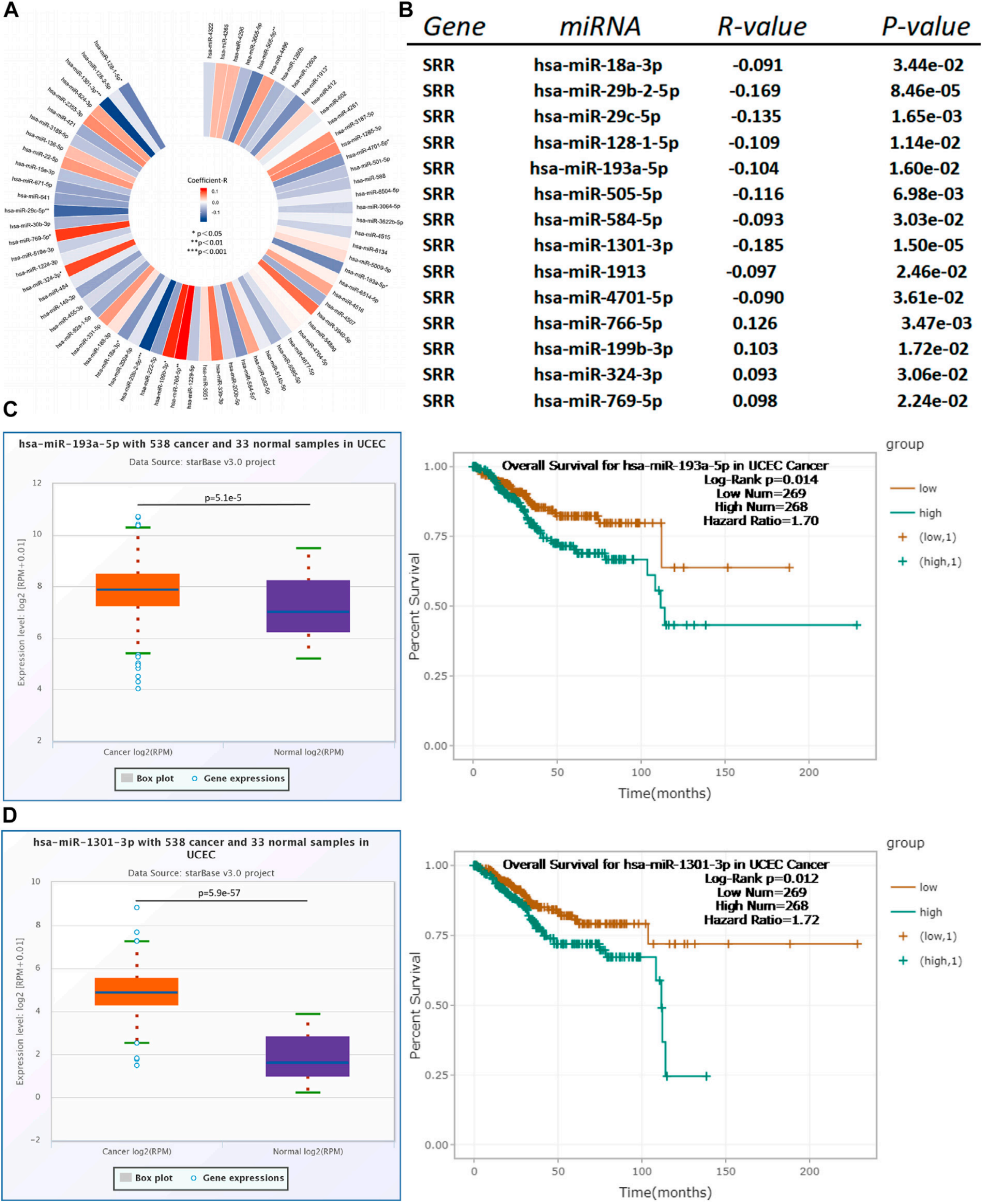
Identification of hsa-miR-193a-5p and hsa-miR-1301-3p as the most potential upstream miRNAs of SRR in UCEC. **(A)** A miRNA-SRR regulatory network in UCEC is constructed. **(B)** A total of 14 miRNAs showing significant correlations with SRR in UCEC are discovered by StarBase. Expression and survival analysis of hsa-miR-193a-5p **(C)** and hsa-miR-1301-3p **(D)** in UCEC patients conducted through StarBase.

MicroRNAs play an important role in regulating gene expression in the human body. They work post-transcriptionally to suppress protein synthesis in most cases (Fabian et al., 2010). We discovered 72 miRNAs after predicting the upstream miRNAs of SRR. We then investigated and visualized their correlations in Figure 10A, based on the hypothesis that there should be a negative relationship between SRR and miRNA expression. With a *p*-value of 0.05, 14 miRNAs could potentially interact with SRR, and 10 were strongly and negatively linked with SRR (Figure 10B, Supplementary Figure S12A).

We then looked at the expression differences in normal and tumor tissues using StarBase miRNA expression data, and we confirmed the results using TCGA. Seven miRNAs, including hsa-miR-18a-3p, hsa-miR-128-1-5p, hsa-miR-193a-5p, hsa-miR-505-5p, hsa-miR-584-5p, hsa-miR-1301-3p, and hsa-miR-1913, showed significant upregulation in tumor tissues (Supplementary Figures S12B,C).

Finally, StarBase was used to determine the prognostic power of the seven miRNAs in UCEC. As presented in Supplementary Figure S12D, only high expression of hsa-miR-193a-5p (HR = 1.70, Log-Rank *p* = 0.014) and hsa-miR-1301-3p (HR = 1.72, Log-Rank *p* = 0.012) was negatively linked with patients’ OS (Figures 10C,D). We used TCGA survival data to verify and confirm the prognostic value of hsa-miR-193a-5p and hsa-miR-1301-3p at OS, DSS, and DFI and plotted Kaplan-Meier curves as presented in Supplementary Figures S12E,F. To conclude, the upstream miRNAs hsa-miR-193-5p and hsa-miR-1301-3p may be suppressing SRR expression in UCEC.

### Prediction of Upstream lncRNAs That May Interact With Hsa-miR-193a-5p or Hsa-miR-1301-3p

The upstream lncRNAs of hsa-miR-193a-5p or hsa-miR-1301-3p were investigated further using StarBase. For hsa-miR-193a-5p and hsa-miR-1301-3p, the total number of predicted lncRNAs was 97 and 153, respectively. After combining the results from StarBase, GEPIA2, and TCGA-UCEC, a total of 13 upstream lncRNAs of hsa-miR-193a-5p were identified, including AC008969.1, LINC00963, C1RL-AS1, XIST, SNHG7, AC008443.1, TTN-AS1, LINC01278, SLC25A21-AS1, AC024075.2, HEIH, AL662795.1, and LINC00294 (Supplementary Figures S13A–C) and 19 lncRNAs of hsa-miR-1301-3p, including MATN1-AS1, RAMP2-AS1, MIR99AHG, SH3BP5-AS1, MBNL1-AS1, AC008443.1, MUC20-OT1, LINC02381, AL590705.5, AC068888.1, AC015712.2, TSPOAP1-AS1, ILF3-AS1, AC012313.3, AC012531.2, AL137058.2, AC015871.3, AL662795.1, and LINC00294 (Supplementary Figures S14A–C) were chosen as being significantly downregulated lncRNAs in UCEC when compared to normal controls.

**FIGURE 11:**
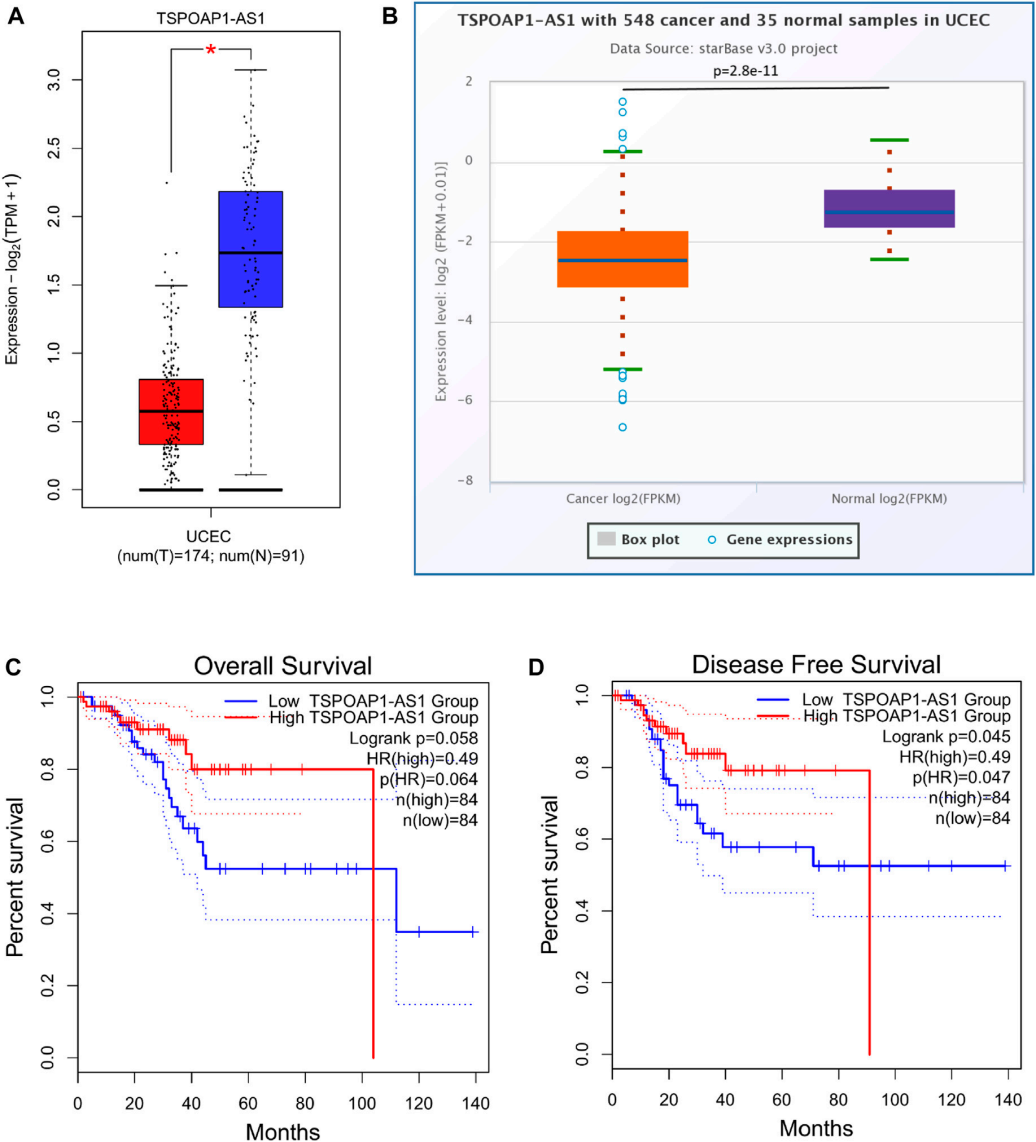
TSPOAP1-AS1 is downregulated in UCEC, while its high expression predicts favorable outcomes. Expression analysis of TSPOAP1-AS1 using GEPIA2 **(A)** and StarBase **(B)**. * indicates *p* < 0.05. Survival analysis of TSPOAP1-AS1 in terms of overall survival **(C)** and disease-free survival **(D)** of UCEC patients.

The prognostic values of the selected lncRNAs were then assessed. None of the predicted 13 lncRNAs of hsa-miR-193a-5p showed significant OS, DSS, or PFI, as shown in Supplementary Table S6. We discovered that only patients with higher TSPOAP1-AS1 expression had better survival outcomes among the 19 lncRNAs of hsa-miR-1301-3p, and the results analyzed through GEPIA2 (Figures 11C,D) were validated in StarBase (Supplementary Figure S14D) and TCGA (Supplementary Figure S14E). Supplementary Table S7 contains detailed prognostic data for the 19 lncRNAs of hsa-miR-1301-3p. GEPIA2 and StarBase were used to compare the expression of TSPOAP1-AS1 in tumor and normal tissues, as shown in Figures 11A,B, respectively.

**FIGURE 12:**
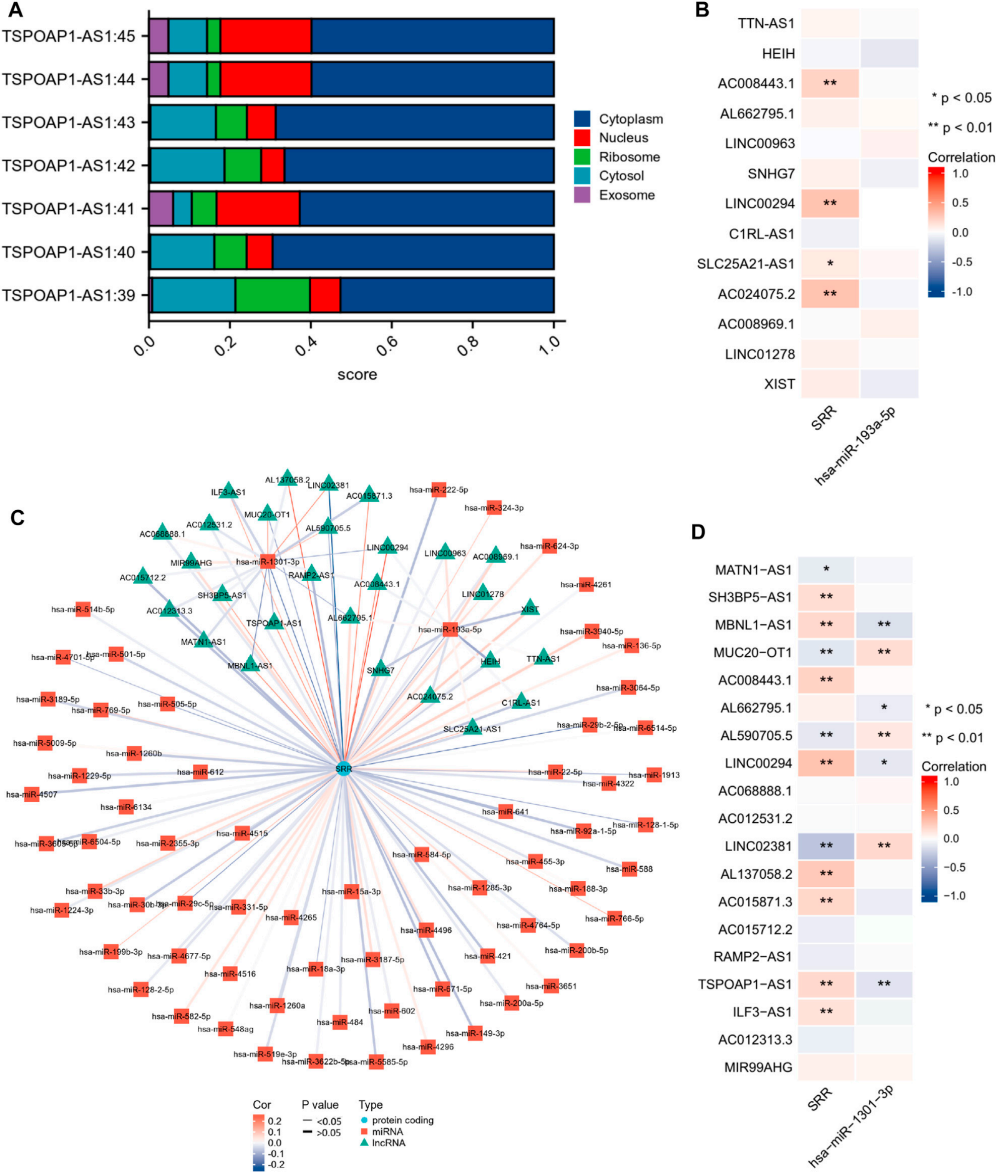
Construction and correlation analysis of the ceRNA network. **(A)** Cellular localization of seven different transcripts of TSPOAP1-AS1 predicted using LncLocator. **(B,D)** Expression correlation analysis of the ceRNA network visualized through a heatmap. *p < 0.05, **p < 0.01. **(C)** LncRNAs-miRNAs-SRR interaction network.

As is well known, lncRNAs frequently act as “sponges” for miRNAs, reducing the miRNA’s suppressive effect on target mRNAs and thus increasing mRNA expression. As a result, lncRNA and miRNA expression will be negatively correlated, while lncRNA and mRNA expression will be positively correlated. We found no significant correlation between hsa-miR-193a-5p and the corresponding 13 lncRNAs (Figure 12B). The expression of TSPOAP1-AS1 was significantly and negatively correlated with hsa-miR-1301-3p. Simultaneously, it was significantly and positively correlated with SRR (Figure 12D). TSPOAP1-AS1 could be the potential upstream lncRNA of hsa-miR-1301-3p in UCEC, based on expression analysis, survival analysis, and correlation analysis.

Finally, because different cellular localizations of lncRNAs determine different mechanisms, we looked at TSPOAP1-AS1’s subcellular location. Figure 12A shows that all seven TSPOAP1-AS1 transcripts were primarily found in the cytoplasm, indicating that TSPOAP1-AS1 could act as a ceRNA to boost SRR expression by competitively sponging hsa-miR-1301-3p. Figure 12C shows a molecular interaction network diagram for better visualization.

## Discussion

UCEC, unlike other cancers, has an increasing incidence and associated mortality (Lu and Broaddus, 2020). Hence, elucidating the underlying mechanisms of UCEC carcinogenesis and discovering new biomarkers help address the rising number of UCEC cases and improve patient outcomes. Through multi-omics analysis, we focused on the function of SRR in EC and found that SRR could be a promising biomarker for accurate diagnosis and targeted therapy.

SRR is an enzyme that catalyzes the conversion of L-serine to D-serine. Many previous studies have been conducted on its role in the central nervous system. According to Rani et al., D-serine acts as a co-agonist of the N-methyl D-aspartate receptor. SRR hyperactivation may cause many neurological disorders. They also showed that incorporating SRR changed the dangerous functions of harmful proteins. It could also produce pyruvate and ammonia after an elimination reaction with L-serine and D-serine (Rani et al., 2020). Only a few studies have recently looked into its potential role in cancer. Ohshima et al. (2020) discovered that SRR promoted colorectal cancer cell proliferation by contributing to the pyruvate pool. In osteosarcoma 143B cells, Gorska-Ponikowska et al. (2017) observed an anticancer effect of high concentrations of glycine and D-serine. SRR’s metabolic activities differ in cancer types, explaining some of these discrepancies.

Our study discovered that SRR expression was low in UCEC, but that higher expression predicted better OS, DSS, and PFI. The expression of SRR decreased as tumor grading and staging increased. Additionally, SRR expression could be an independent predictor of OS, DSS, and PFI. All this suggested that SRR plays a protective role in UCEC. According to our GSEA enrichment results, SRR expression was linked to many metabolic pathways, including fatty acid degradation and fructose metabolism, lipoic acid metabolism, valine, tyrosine, and leucine isoleucine. Furthermore, our GO enrichment analysis revealed that SRR was linked to various cell cycle and DNA replication-related processes, which had previously been suggested as prospective targets for the precision treatment of patients with EC (Lheureux and Oza, 2016). As a result, it is not difficult to believe that SRR expression influences many critical metabolic and DNA replication pathways involved in EC cell proliferation and migration.

The existence of L-isomers of the most nutritionally important amino acids in the human body is widely acknowledged. When D-amino acid (the mirror-image enantiomer of L-amino acid) is substituted in a protein, the protein’s function and structure are altered (Pundir et al., 2018). Many previous studies have reported L-serine’s carcinogenic effect in cancers, such as its proliferative effect on breast cancer cells (Pollari et al., 2011; Amelio et al., 2014; Yang and Vousden, 2016). L-serine deficiency also increased drug sensitivity in lymphoma, leukemia, and liver cancers (Maddocks et al., 2017). As previously stated, SRR is involved in the metabolism of L-serine, and the decrease of L-serine may result in a reduced one-carbon metabolism source, which has been linked to tumor growth (Amelio et al., 2014; Newman and Maddocks, 2017). This result is consistent with our GSEA, which revealed that SRR was negatively correlated with the one-carbon pool. Moreover, SRR has been linked to glucose homeostasis in peripheral tissues. Because of the lack of synthesized D-serine, Lockridge et al. (2016) claimed that SRR knockout mice secreted more insulin. Similarly, Suwandhi et al. (2018) reported that chronic D-serine supplementation reduced insulin secretion, affecting systemic glucose metabolism. Insulin resistance and hyperinsulinemia are important events that occur at the start of hyperplasia, and they can trigger EC (Papatla et al., 2016). Insulin promoted EC growth and progression *in vivo* by activating the InsR/IRS-1/PI3K-Akt pathway. By activating the PI3K/Akt pathway, insulin stimulation may enhance cancer cell proliferation and inhibit apoptosis *in vivo* (Wang et al., 2012; Tian et al., 2017). According to a comprehensive systematic review and meta-analysis, higher fasting insulin was also linked to EC (Hernandez et al., 2015). We hypothesized that the higher level of SRR, the more L-serine was converted to D-serine. On the one hand, lower L-serine levels in cancerous endometrial tissue meant a low risk of cancer; on the other hand, higher D-serine levels in tumor tissue could regulate glucose homeostasis, preventing the activation of key cancer pathways.

SRR was primarily involved in RNA modification, ferroptosis, and DNA damage repair processes in our enrichment analyses. RNA modifications are gaining more attention these days, and mounting evidence suggests that disruption of RNA epigenetic processes plays a role in developing human illnesses like cancer (Barbieri and Kouzarides, 2020). According to our findings, in most cases, SRR and RNA modification genes were strongly and positively linked in UCEC. The SRR-high expression group had a more active m6A modification situation. According to Liu et al. (2018), EC had low levels of m6A mRNA methylation, and that reduced m6A methylation promoted cancer cell proliferation. We could reasonably conclude that SRR plays a role in the positive regulation of m6A RNA methylation in EC, thereby exerting its anticancer effects. Ferroptosis is a non-apoptotic, novel type of programmed cell death that serves as an adaptive mechanism for eliminating malignant cells, and it represents a new pathway for tumor suppression (Li et al., 2020; Fan et al., 2021a). Previous studies have found a link between ferroptosis and the growth and proliferation of UCEC. According to Janeiro et al., ferroptosis was dysregulated in low-grade, early-stage EC (López-Janeiro et al., 2021). Wang et al. (2021) discovered that silencing PTPN18 promoted ferroptosis, decreased proliferation, and induced apoptosis in KLE cells by targeting the p-P38/GPX4/xCT axis. Furthermore, Zou et al. (2020) and Kuganesan et al. (2021) found that the peroxisome and p53 were crucial for ferroptosis sensitization in EC cells (Liu and Gu, 2021). SRR was positively correlated with the peroxisome, the p53 signaling pathway, and the ferroptosis process, and the SRR-high expression group had upregulated ferroptosis-related genes. As a result, we reasoned that SRR might positively regulate peroxisome and p53 signaling in UCEC, causing an active ferroptosis state and suppressing tumor cell biological behavior. DNA damage repair includes a variety of mechanisms that are essential to genome integrity and proper function (Jinjia et al., 2019). Cancer cells have a lower capacity for DNA repair and DNA damage signaling than normal cells, and cancer can upregulate DNA repair pathways and drive tumorigenesis in certain circumstances (Brown et al., 2017). Moreover, it was reported that the ability to identify and repair DNA mismatches contributed to better outcomes in patients with EC. In contrast, the loss of DNA mismatch repair was linked to adverse outcomes (Cohn et al., 2006). In our study, SRR expression was significantly and positively correlated with most DNA damage repair-related genes in patients with UCEC, indicating that SRR is likely to play a role in the DNA damage repair process, contributing to favorable prognoses. Positive correlations between SRR and many other tumor suppressor genes were discovered in UCEC, indicating that they may act synergistically as cancer inhibitors.

Immune cells and cytokines can be found in large numbers in EC tissues, stimulating an endogenous antitumor immune response (Cao et al., 2021). In our study, SRR expression was significantly and positively correlated with the levels of CD8^+^ cytotoxic T cells and dendritic cell infiltration. Dendritic cells in EC were found to phagocytize and process tumor-associated antigens, resulting in a CD8^+^ T cells response that killed EC cells directly (Chen et al., 2020). Meanwhile, studies have shown that CD8+T cells and dendritic cells have tumor-suppressing and survival-enhancing properties (De Felice et al., 2019; Li and Wan, 2020; Wang G et al., 2020; Rousset-Rouviere et al., 2021). This suggested that SRR was important in regulating tumor immunity and, therefore, influenced patient prognoses.

We then looked at using SRR as a marker for chemotherapy and immune therapy in patients with UCEC to see if it could be used in clinical treatment. Immunotherapy is more likely to benefit EC than other types of gynecological malignancies (Cao et al., 2021). TMB and MSI were predictive markers for immune checkpoint inhibitors. It was widely assumed that higher TMB and MSI indicated better immunotherapy response (Schrock et al., 2019; Mazloom et al., 2020; Salem et al., 2020). In the present study, SRR was found to have positive correlations with TMB and MSI in UCEC. Furthermore, the correlations between SRR and some immune inhibitors, such as PDCD1 and CTLA4, which have been reported to enhance the immune responses, were striking (Fan et al., 2021b; Lu et al., 2022). Immune checkpoint expression also differed between the SRR-high and SRR-low expression groups. These findings suggested that SRR could be used to predict immunotherapeutic response. Doxorubicin, docetaxel, paclitaxel, and cisplatin have become popular in the treatment of advanced and recurrent endometrial cancer (Brooks et al., 2019; Nomura et al., 2019). We discovered that the IC50 of doxorubicin, docetaxel, and paclitaxel was higher in the SRR-low expression group than in the SRR-high expression group, implying that patients with SRR-high expression were more sensitive to these drugs. Because chemotherapy drugs can have serious adverse effects, it is vital to screen people who are sensitive to them so that adverse reactions are minimized (Fan et al., 2021b; Lu et al., 2022). SRR expression could also be used as a biomarker to screen patients with UCEC for chemotherapy, according to our findings.

Four distinct molecular subgroups with prognostic significance were previously identified in the genetic landscape mapping of patients with EC. POLE-ultramutated, MSI-hypermutated, copy-number low, and copy-number high were all found in them. The first group exhibited the best PFS, followed by the MSI-hypermutated group, and patients with a high copy number had the worst PFS (Le Gallo and Bell, 2014; Auguste et al., 2018). Many other studies were consistent with the above view (Church et al., 2015; Van Gool et al., 2015; Mcconechy et al., 2016; Bell and Ellenson, 2019; Imboden et al., 2019). SRR expression was higher in the POLE-ultramutated and MSI-hypermutated groups in our study. In contrast, it was lowest in the copy number-high group, confirming the link between SRR expression and UCEC patient survival. Thorsson et al. (2018) also identified six immune subgroups spanning multiple tumor groups, including EC, based on differences in macrophages or lymphocytes. Patients in the C3 (inflammatory) subgroup had the best prognoses. In contrast, those in the C2 (INF-gamma dominant) and C1 (wound healing) subgroups had less favorable outcomes, and those in the C4 (lymphocyte depleted) and C6 (TGF-b dominant) subgroups had the worst outcomes (Thorsson et al., 2018; Mullen and Mutch, 2019). We found that SRR was significantly higher in the C3 group and significantly lower in the C4 group, indicating that SRR may influence the tumor microenvironment and benefit patient survival.

In terms of somatic mutations in UCEC, our study found that patients in the SRR-low group had lower PTEN, PIK3CA, TTN, and ARID1A mutation frequencies, while having a higher TP53 mutation frequency. Liu J et al. (2020) previously identified a cell cycle-related signature in patients with UCEC, finding that samples with high risk scores (poor survival outcomes) had lower mutation rates of PTEN, TTN ARID1A, and PIK3CA and a higher mutation rate of TP53. This was nearly identical to our findings. The discovery that ARID1A and TP53 may cooperate in a complex system could explain why TP53 mutations were mutually exclusive with ARID1A (Wang et al., 2011; Bosse et al., 2013; Wu et al., 2014). Additionally, activating PIK3CA mutations were frequently found alongside PTEN mutations (Cheung et al., 2011). Furthermore, PTEN mutation was associated with a better prognosis than the PTEN non-mutation group (Tao and Liang, 2020). These perspectives may offer plausible explanations for the difference in mutation frequencies of specific genes between SRR-high and SRR-low expression groups.

MicroRNAs repress multiple genes at the mRNA and translation level, which is how they perform their biological function. Our study discovered that hsa-miR-193a-5p and hsa-miR-1301-3p could be potential miRNAs upstream of SRR. Previous studies have suggested that hsa-miR-193a-5p may play a role in the invasiveness of malignant pleural mesothelioma cells (Jotatsu et al., 2020), and a significant reduction in hsa-miR-193a-5p level was observed after irradiation of the colorectal cancer cell line HCT116 (Yu et al., 2021). Similarly, Pu et al. (2016) reported that hsa-miR-193a-5p contributes to osteosarcoma metastasis by suppressing SRR expression. Another candidate miRNA, hsa-miR-1301-3p, was upregulated in early-stage nasopharyngeal carcinoma (Zheng et al., 2021). However, no studies have investigated the target genes and mechanisms of both hsa-miR-1301-3p and hsa-miR-193a-5p in the context of UCEC. The potential lncRNAs of the hsa-miR-193a-5p/SRR or hsa-1301-3p/SRR axis should be antineoplastic in UCEC, according to the ceRNA hypothesis, and the most eligible one turned out to be TSPOAP1-AS1. Zheng et al. (2020) identified TSPOAP1-AS1 as protective against cervical cancer. Tang et al. (2021) and Giulietti et al. (2018) reported that higher TSPOAP1-AS1 expression in pancreatic cancer was associated with a better prognosis. TSPOAP1-AS1 plays a significant role in LUAD, READ, and THYM. While in UCEC, they calculated the HR to be 0.441 and the *p*-value to be 0.176 after dividing the patients into two groups with the median expression of TSPOAP1-AS1, which was not entirely consistent with our results. It is possible that different datasets were used in the different studies as an explanation. However, TSPOAP1-AS1 increased VEGFA expression and accelerated tube formation in hepatocellular carcinoma cells, promoting angiogenesis (Wang et al., 2019). To summarize, our findings suggest that hsa-miR-193a-5p and hsa-miR-1301-3p may regulate SRR expression in UCEC and that the TSPOAP1-AS1/hsa-miR-1301-3p/SRR axis could be a promising therapeutic target. Regardless, the detailed mechanism needs to be investigated further.

Our study does, without a doubt, have some limitations. Due to the lack of prognostic information on patients with UCEC in other datasets, such as GEO and the International Cancer Genome Consortium, we could not use external datasets to validate the survival results. Additionally, *in vivo* and *in vitro* studies are required to confirm our findings.

In summary, we show that SRR expression is low in many cancer types, including UCEC, and that higher SRR expression in patients with UCEC indicates a better prognosis. Additionally, SRR expression raises immune infiltration in patients with UCEC. SRR expression is linked to some immune checkpoints and TMB and MSI scores, suggesting that it may influence the immune microenvironment and serve as a therapeutic target for patients with UCEC. Furthermore, our findings indicate that SRR may inhibit cancer by activating ferroptosis, m6A methylation, and DNA-damage repair processes. Finally, the hsa-miR-193a-5p/SRR axis and TSPOAP1-AS1/hsa-miR-1301-3p/SRR axis are the most likely regulatory targets of SRR. Our discussion spans all the possible mechanisms in great detail. Nevertheless, fundamental investigations and thorough clinical trials will be required in the future to confirm our findings.

## Data Availability

Publicly available datasets were analyzed in this study. These data can be found freely from TCGA data portal (https://portal.gdc.cancer.gov/) and GEO database (https://www.ncbi.nlm.nih.gov/geo/).

## Supplementary Material

Supplementary Figure S1 Flowchart depicting the workflow of this study.

Supplementary Figure S2
**(A)** Expression of SRR in different cell lines. The abscissa represents different groups of cancer cells, and the ordinate represents the expression distribution of SRR expression. **(B)** SRR RNA expression in HPA normal tissues (data from GTEx). **(C)** SRR RNA expression in HPA cancer tissues (data from TCGA).

Supplementary Figure S3 Prognostic value of SRR in UCEC. **(A–C)** 2-year, 5-year, and 10-year OS in the UCEC cohort. **(D–F)** 2-year, 5-year, and 10-year RFS in the UCEC cohort. **(G)** Differential SRR protein expression between UCEC tissues and normal tissues using the UALCAN database.

Supplementary Figure S4
**(A)** Correlation between the infiltration of six immune cells and the prognoses of patients with UCEC. **(B)** Correlation between somatic copy number alterations and immune cell infiltration level. **(C)** Comparison of the expression of immune checkpoint-related genes between UCEC tumor (red) and normal (blue) tissues. **(D)** SRR expression in different immune subtypes. **(E)** SRR expression in different molecular subtypes.

Supplementary Figure S5
**(A)** Predicted structural proteins crucial for the functioning of SRR generated from GeneMANIA. Different colors of connecting lines denote the diverse functions. **(B)** The top 50 genes most positively associated with SRR are shown in a heatmap. **(C)** The top 50 genes most negatively associated with SRR are shown in a heatmap.

Supplementary Figure S6
**(A)** Spearman correlation analysis of SRR expression with DNA damage repair-related genes in TCGA UCEC patients. **p* < 0.05, ***p* < 0.01, ****p* < 0.001. **(B)** Spearman correlation analysis of SRR expression with 30 tumor suppressor genes in UCEC and pan-cancer using TIMER2.0 database.

Supplementary Figure S7 Distribution of m6A methylation **(A)** and ferroptosis **(B)**-related genes in UCEC SRR-high expression (group 2, color red) and SRR-low expression (group 1, color blue) groups. The expression value of each gene is converted by z-score, with red representing high expression and blue representing low expression. **p* < 0.05, ***p* < 0.01, ****p* < 0.001.

Supplementary Figure S8 Copy number variation (CNV) analysis of SRR in UCEC. **(A,B)** Bubbles represent the percentage of homozygous and heterozygous CNV in patients with UCEC. The bubble size positively correlates with percentage. Blue represents deletion, and red represents amplification. **(C)** Bubble plot representing the SRR expression profile at the mRNA expression and CNV level. Red bubbles represent positive correlations. The deeper the color, the higher the correlation. Bubble size positively correlates with FDR significance. The black outline border indicates FDR ≤ 0.05. **(D)** Survival difference between CNV groups. Bubble color and size represent the Log-rank *p*-value. The bubble color represents the significance of the Log-rank *p*-value, and the bubble size positively correlates with the significance of the Log-rank *p*-value. The black outline border indicates Log-rank *p*-value ≤ 0.05.

Supplementary Figure S9 Comparative mutation and methylation analysis of SRR. **(A)** The horizontal axis represents the UCEC SRR-low and SRR-high expression groups. The vertical axis represents the gene mutation distribution, and different colors represent different mutation types. **(B)** The correlation between SRR expression and DNA methylation of the SRR promoter region from MEXPRESS in UCEC. The correlation coefficient and *p*-value on the right indicate the relationship between SRR expression and DNA methylation of the promoter. **p* < 0.05, ****p* < 0.001. **(C)** The Kaplan-Meier survival associated with the promoter region cg02945294. Blue represents lower methylation status, and red represents higher methylation status.

Supplementary Figure S10 Associations between SRR expression and clinical stage **(A)** and grade **(C)** across human cancers. The horizontal coordinates indicate different tumor types, and the vertical coordinates indicate the *p*-value after −Log10 conversion. Red color shows significant and negative correlation, while dark shows significant and positive correlation. **(B)** Higher SRR expression is associated with a lower stage in UCEC. **(D)** Higher SRR expression is associated with a lower grade in UCEC.

Supplementary Figure S11
**(A)** Validation of the multivariate cox regression model. The risk score of each sample is ranked from smallest to largest, with red and blue colors representing the high and low groups of risk score. Blue circle shows a living patient, while red triangle indicates death. **(B)** Risk score-based survival analysis of UCEC patients, with red line denoting high-risk score group and blue denoting low-risk score group. Forest plots showing the results of the multivariate Cox regression analysis of clinicopathological characteristics affecting the DSS **(C)** and PFI **(D)** of UCEC patients.

Supplementary Figure S12 Expression, correlation, and survival analysis of the upstream miRNAs of SRR. **(A)** Correlation analysis of the 10 miRNAs showing significantly negative correlations with SRR in UCEC by StarBase. **(B)** Seven out of the 10 miRNAs are significantly highly expressed in UCEC compared with normal tissues through StarBase. **(C)** Validation of the expression differences of the 10 miRNAs by analyzing data obtained from TCGA. **p* < 0.05, ***p* < 0.01, ****p* < 0.001. **(D)** OS analysis of the seven overexpressed miRNAs in UCEC patients by StarBase. OS, DSS, and PFI analysis of hsa-miR-1301-3p **(E)** and hsa-miR-193a-5p **(F)** through TCGA.

Supplementary Figure S13 Expression analysis of the upstream lncRNAs of hsa-miR-193a-5p. Expression analysis of the consistently predicted 13 significantly downregulated upstream lncRNAs of hsa-miR-193a-5p by StarBase **(A)**, GEPIA2 **(B)**, and TCGA **(C)**. **p* < 0.05, ***p* < 0.01, ****p* < 0.001.

Supplementary Figure S14 Expression analysis of the upstream lncRNAs of hsa-miR-1301-3p. Expression analysis of the consistently predicted 19 significantly downregulated upstream lncRNAs of hsa-miR-1301-3p by StarBase **(A)**, GEPIA2 **(B)**, and TCGA **(C)**. **p* < 0.05, ***p* < 0.01, ****p* < 0.001. **(D)** OS analysis of TSPOAP1-AS1 in StarBase. **(E)** OS, DSS, PFI analysis of TSPOAP1-AS1 through TCGA.

Supplementary Table S1 Correlation analysis between SRR expression and clinicopathological variables in EC based on TCGA database.

Supplementary Table S2 SRR expression correlates with clinicopathological characteristics through logistic regression. The lower part shows full forms of all abbreviations in this article.

Supplementary Table S3 Univariate and multivariate analyses of factors influencing patients’ OS in UCEC.

Supplementary Table S4 Univariate and multivariate analyses of factors influencing patients’ DSS in UCEC.

Supplementary Table S5 Univariate and multivariate analyses of factors influencing patients’ PFI in UCEC.

Supplementary Table S6 Survival analysis of the upstream lncRNAs of hsa-miR-193a-5p.

Supplementary Table S7 Survival analysis of the upstream lncRNAs of hsa-miR-1301-3p.
