# Supplementary figures and images for: Integrated Bioinformatics Analysis of Serine Racemase as an Independent Prognostic Biomarker in Endometrial Cancer
Source: Front Genet. 2022 Jul 18;13:906291. doi: 10.3389/fgene.2022.906291 (PMC9340001; doi:10.3389/fgene.2022.906291)

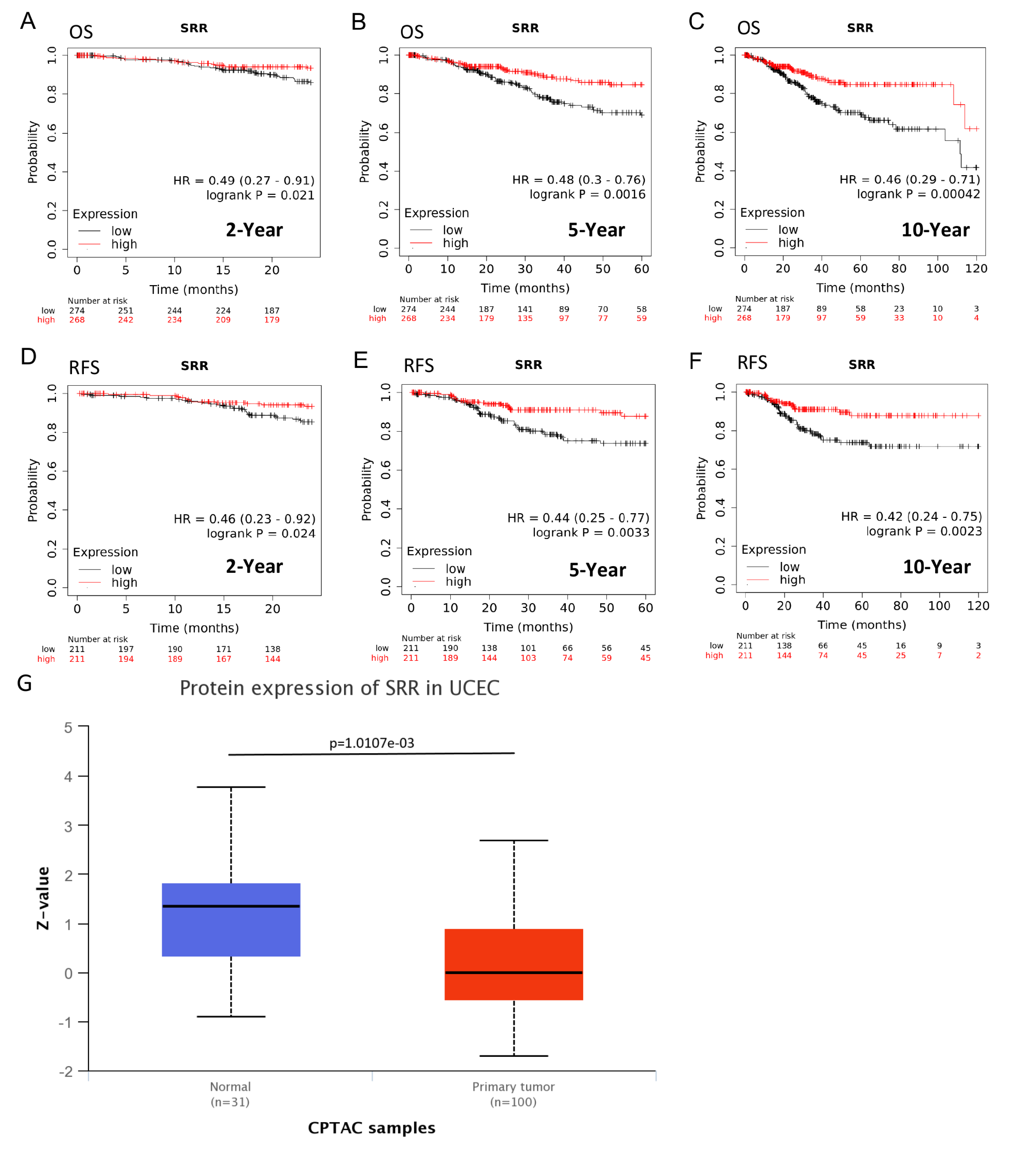

Supplement: Supplementary file 1 [file Image3.TIFF]

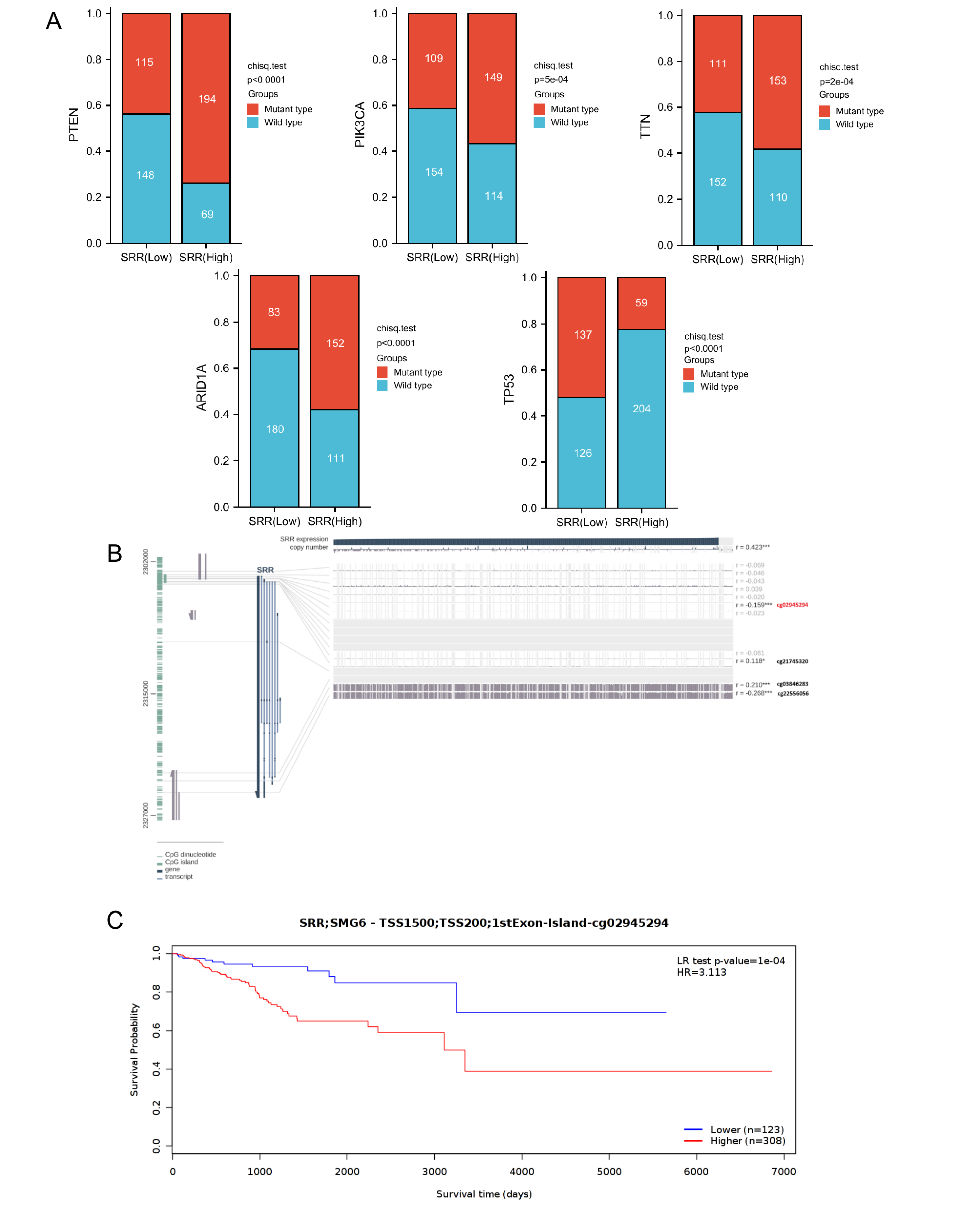

Supplement: Supplementary file 2 [file Image9.TIFF]

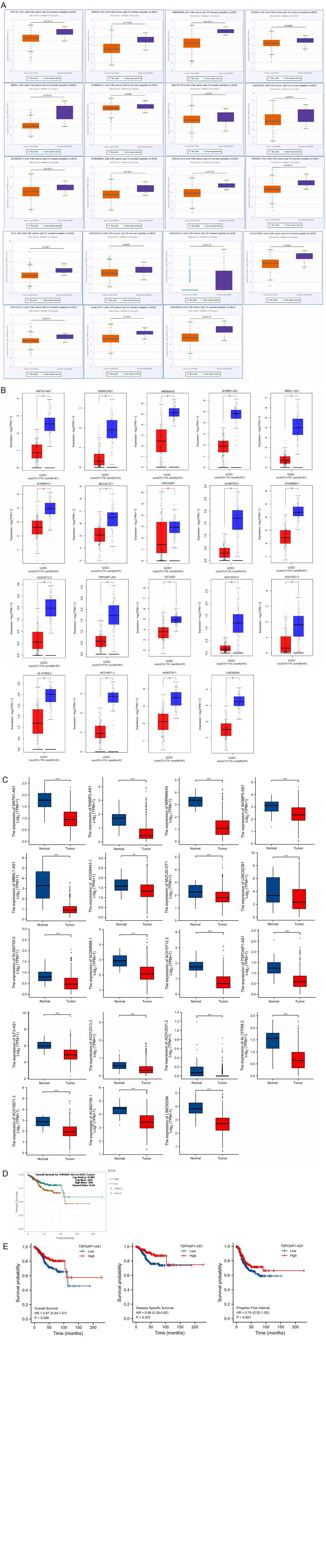

Supplement: Supplementary file 4 [file Image14.TIF]

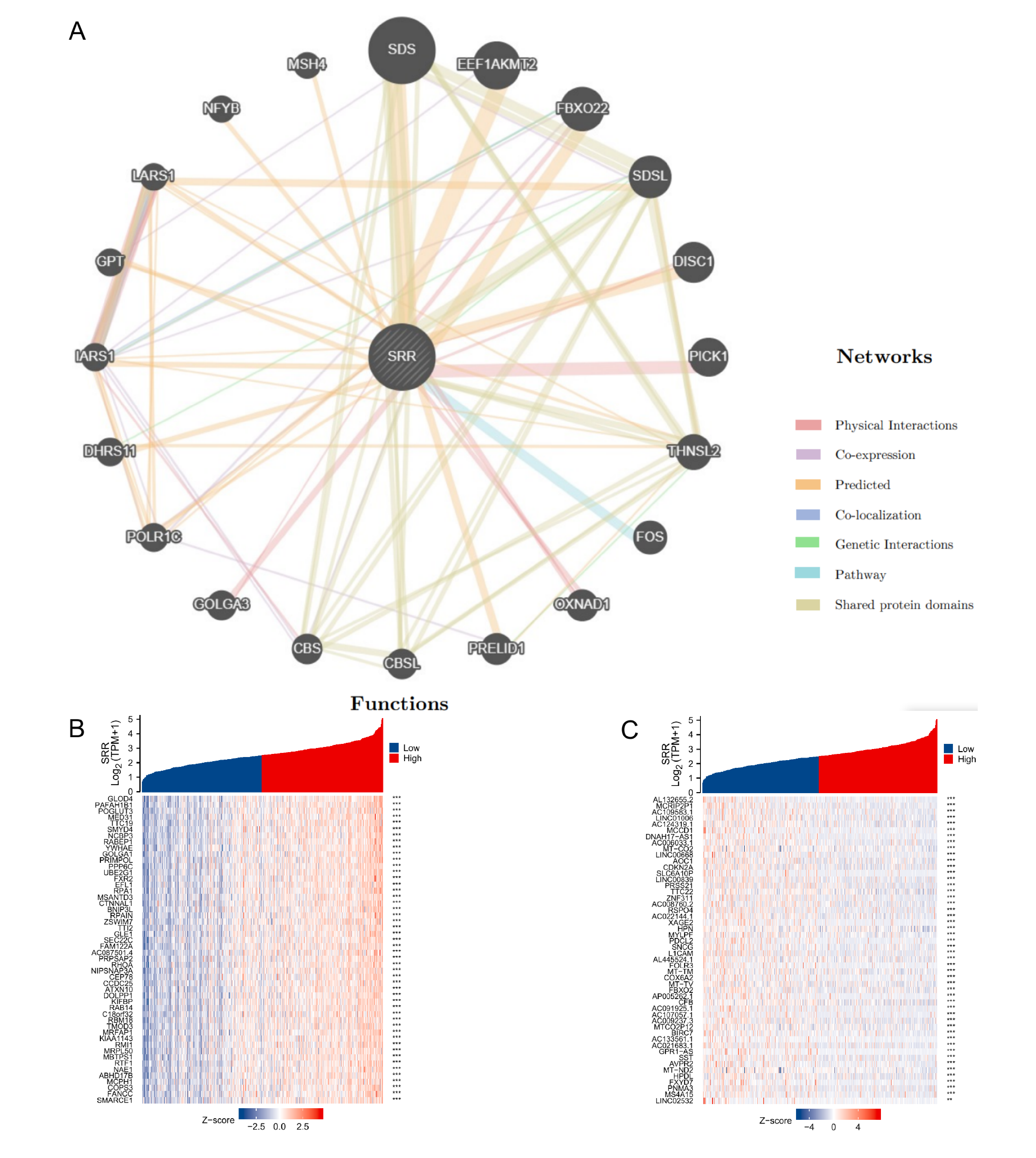

Supplement: Supplementary file 5 [file Image5.TIFF]

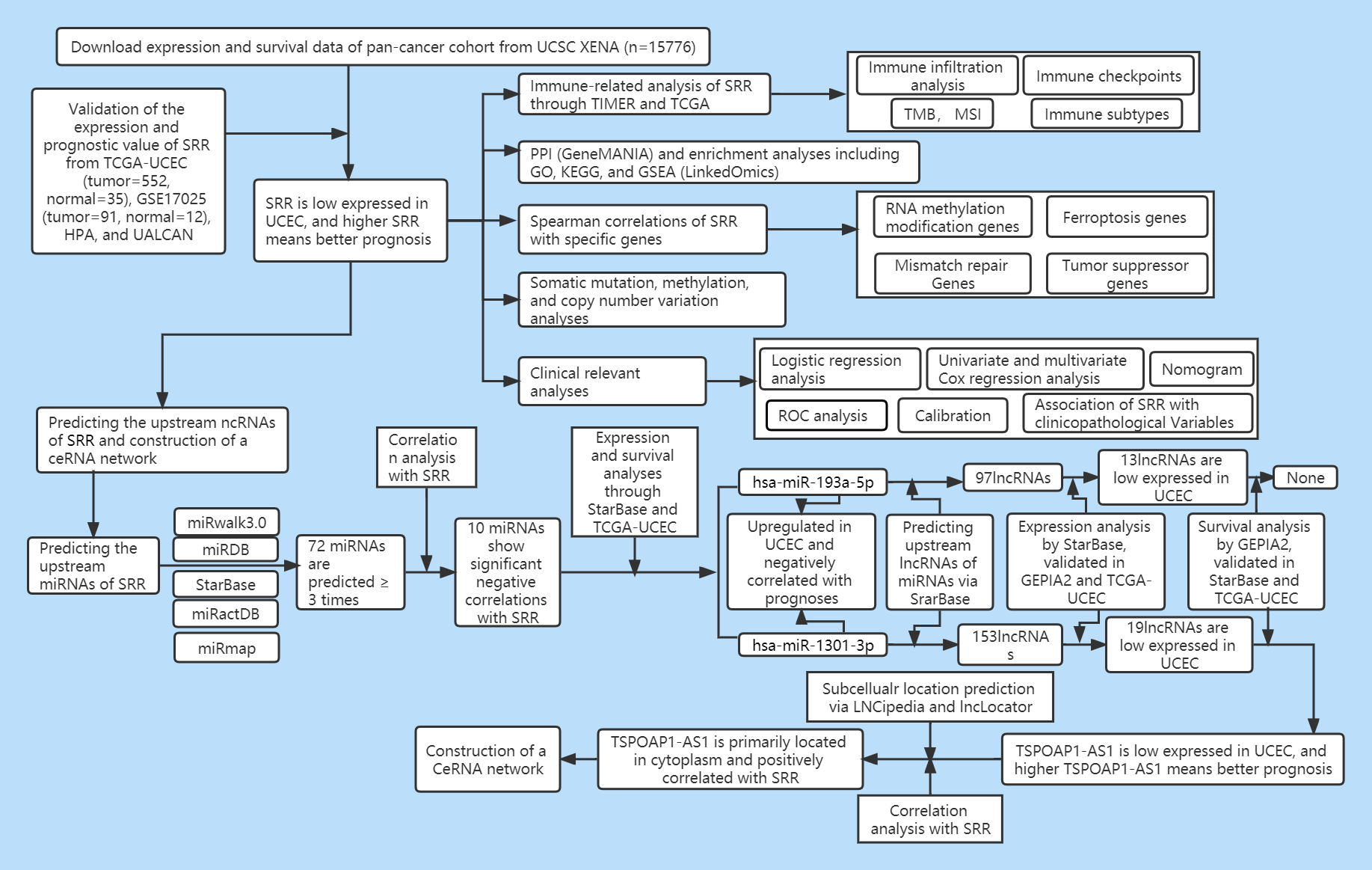

Supplement: Supplementary file 6 [file Image1.JPEG]

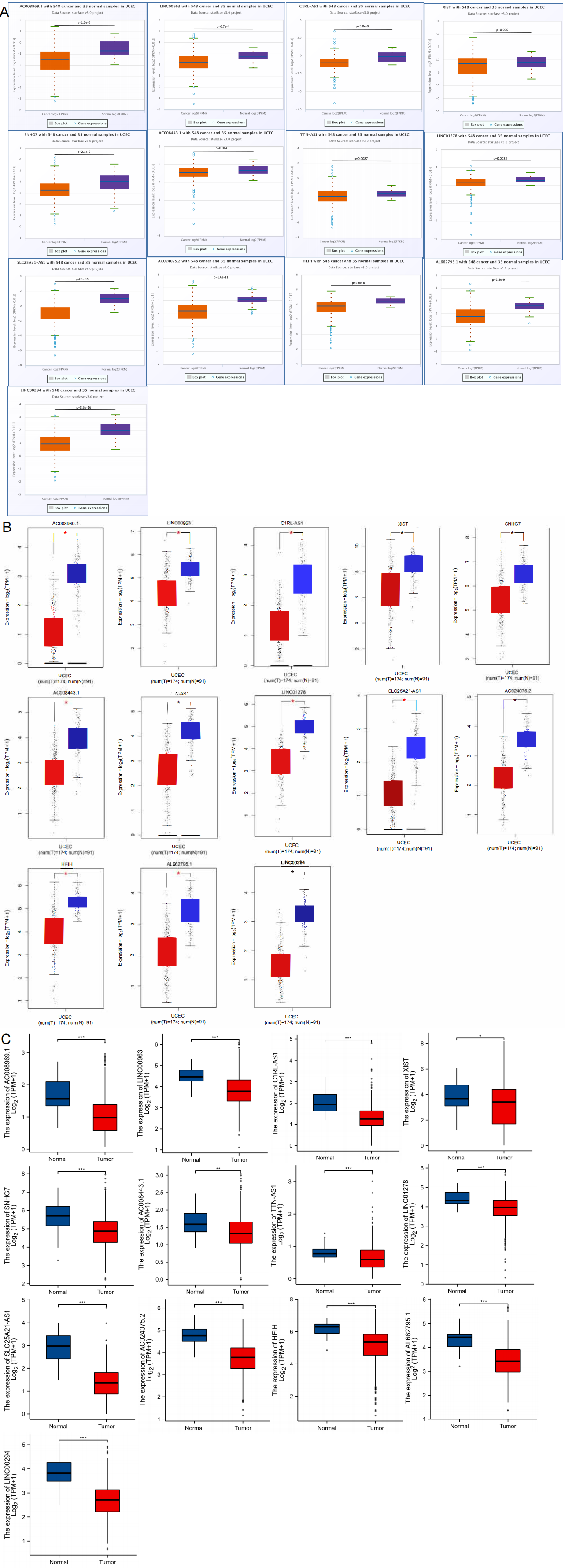

Supplement: Supplementary file 8 [file Image13.TIF]

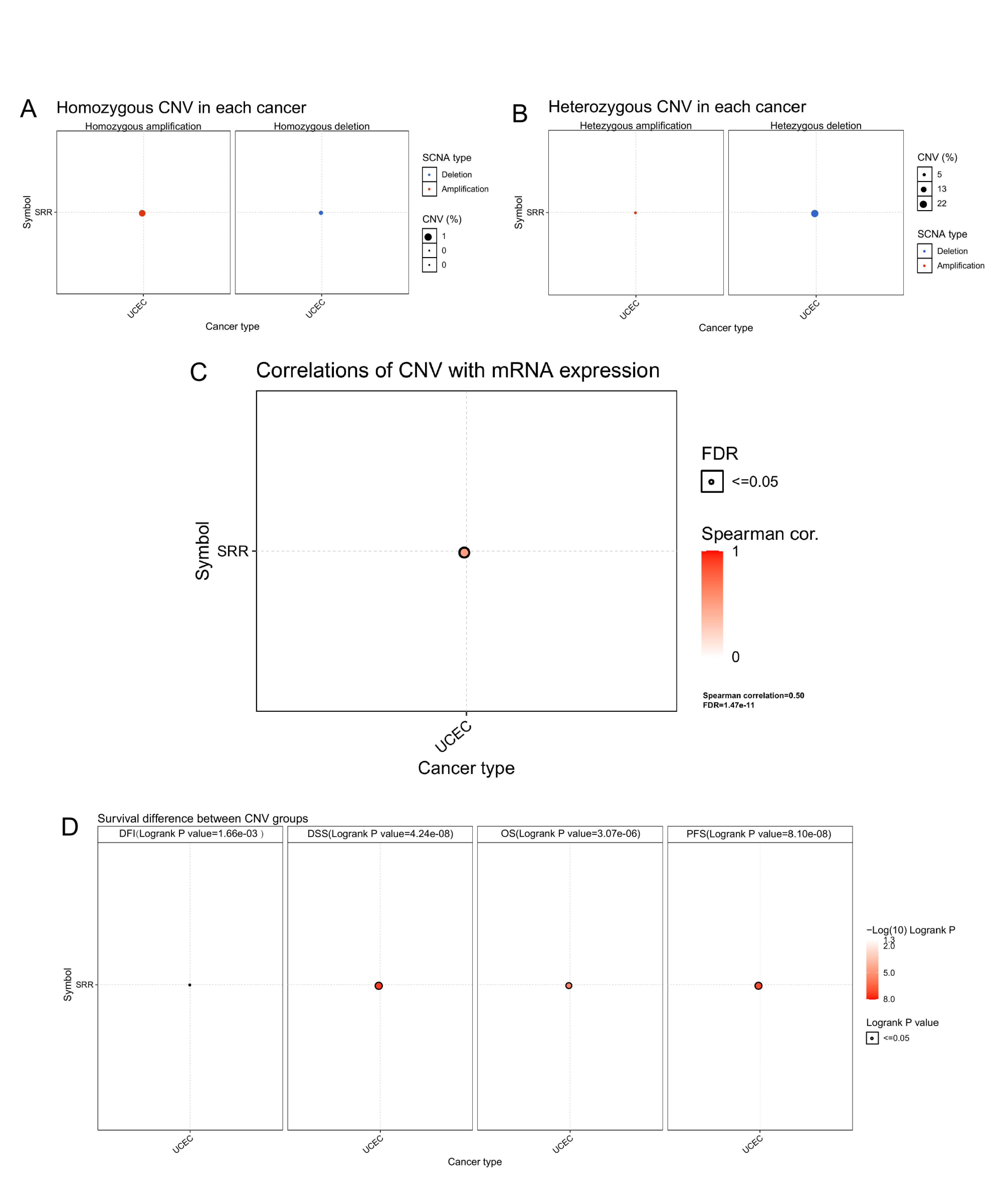

Supplement: Supplementary file 9 [file Image8.TIFF]

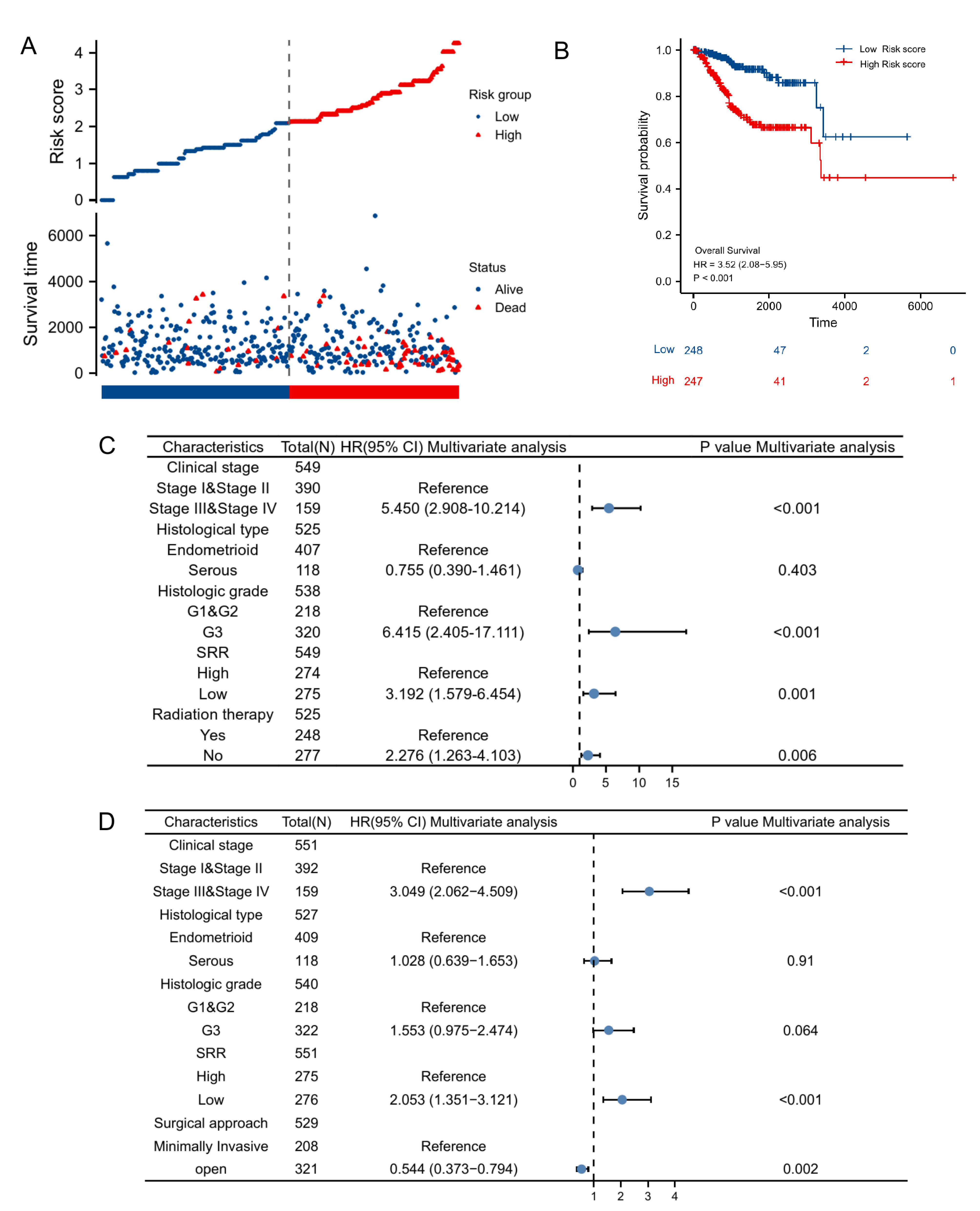

Supplement: Supplementary file 13 [file Image11.TIFF]

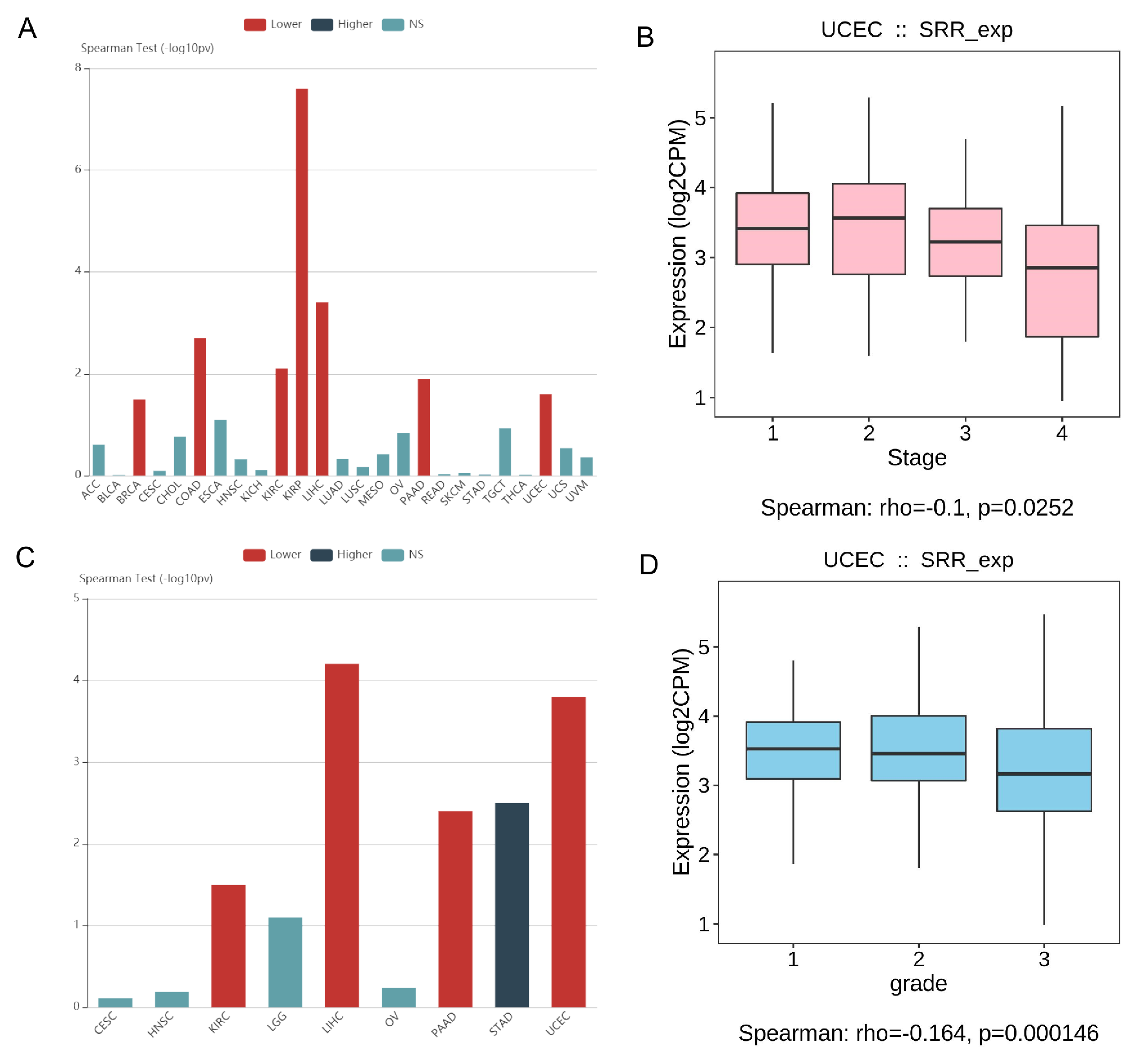

Supplement: Supplementary file 14 [file Image10.TIFF]

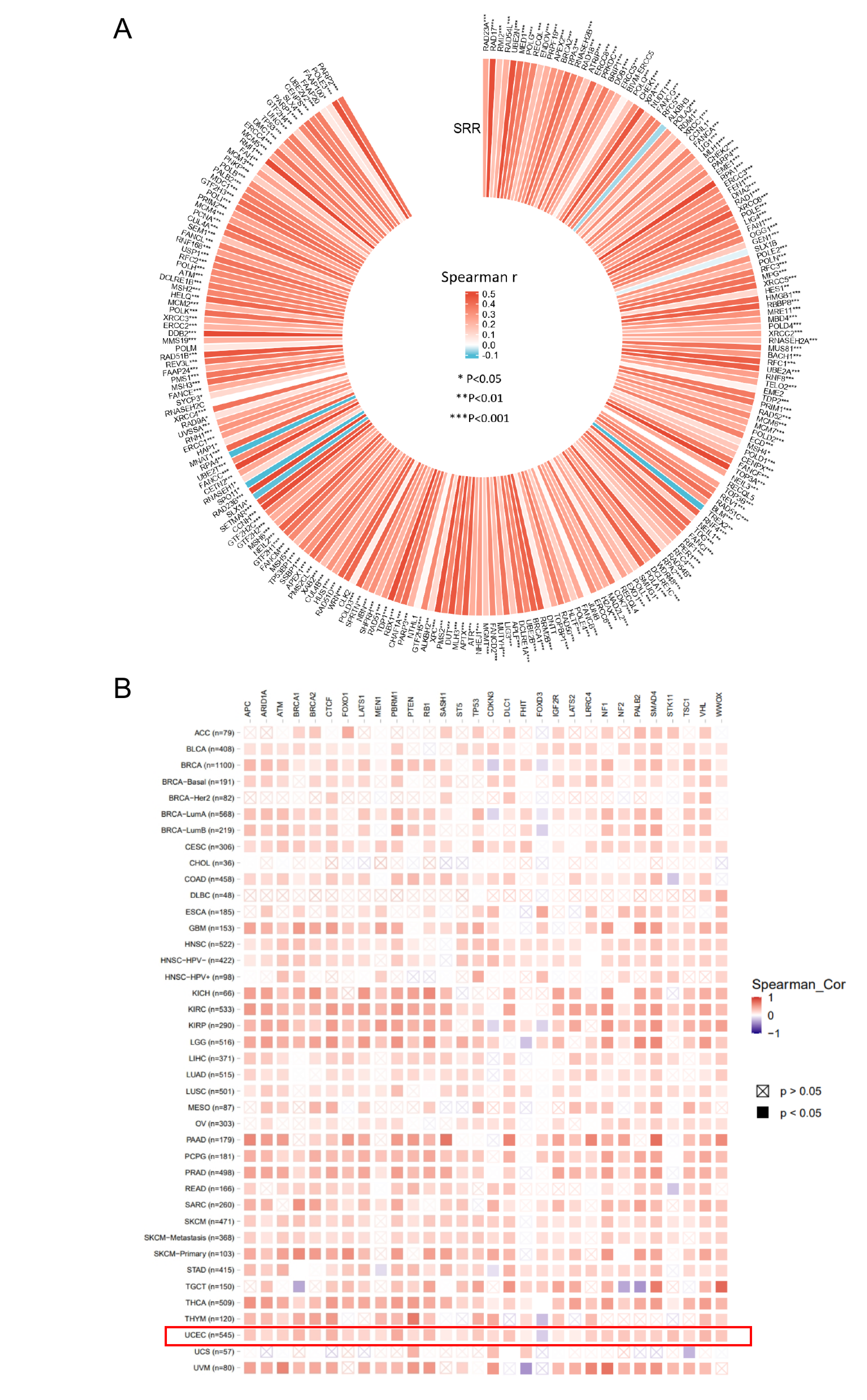

Supplement: Supplementary file 16 [file Image6.TIFF]

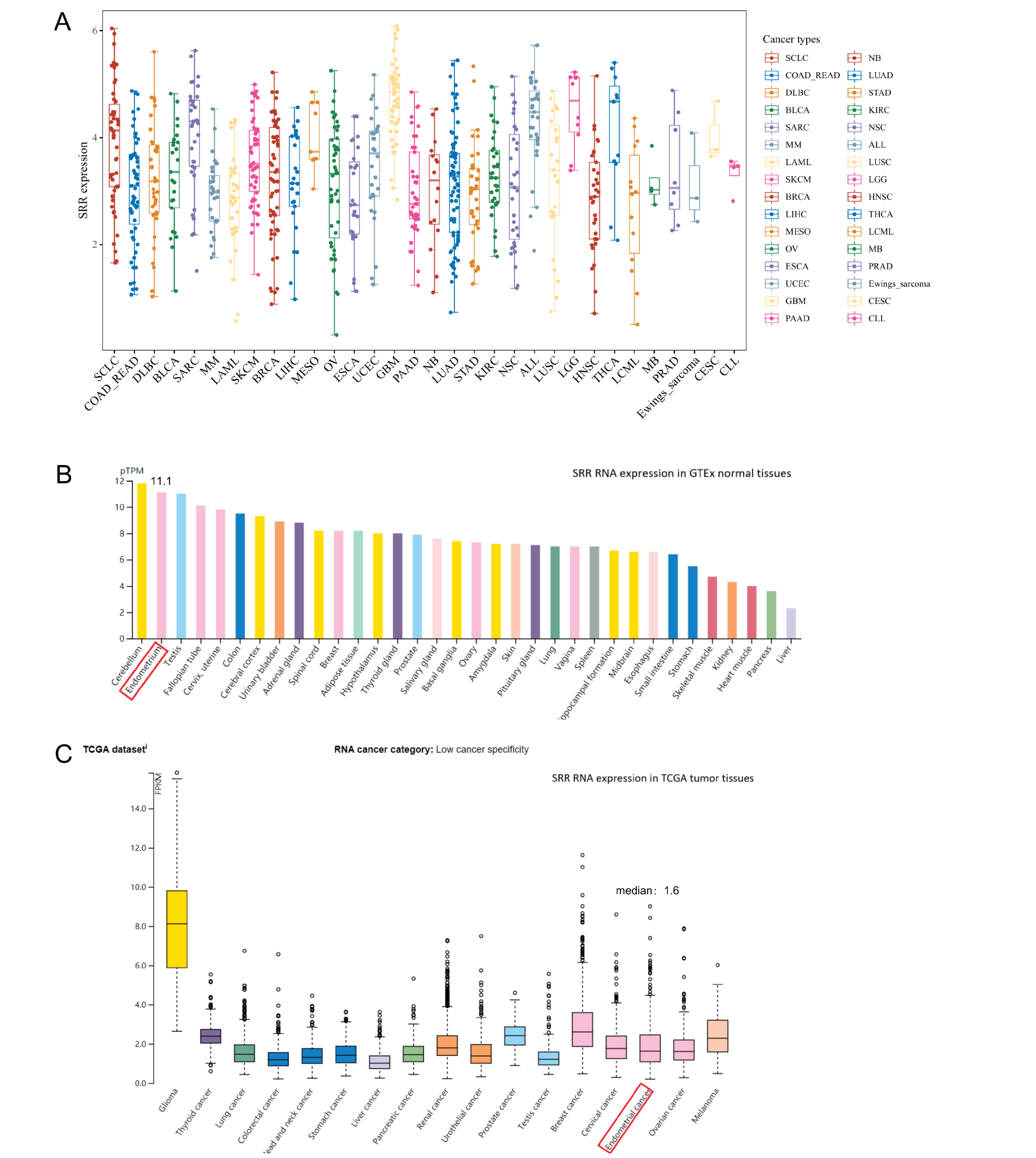

Supplement: Supplementary file 18 [file Image2.TIFF]

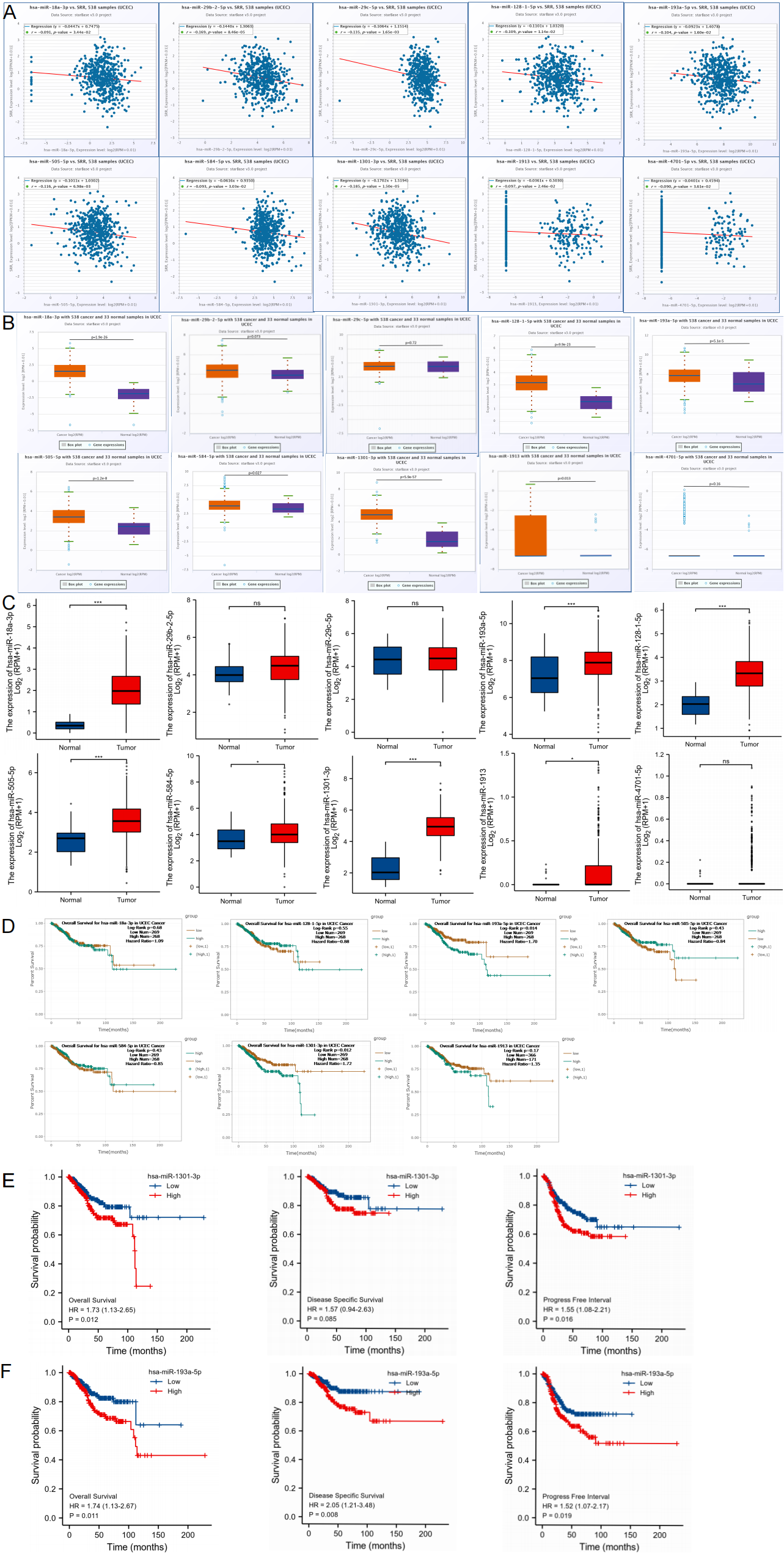

Supplement: Supplementary file 20 [file Image12.TIF]

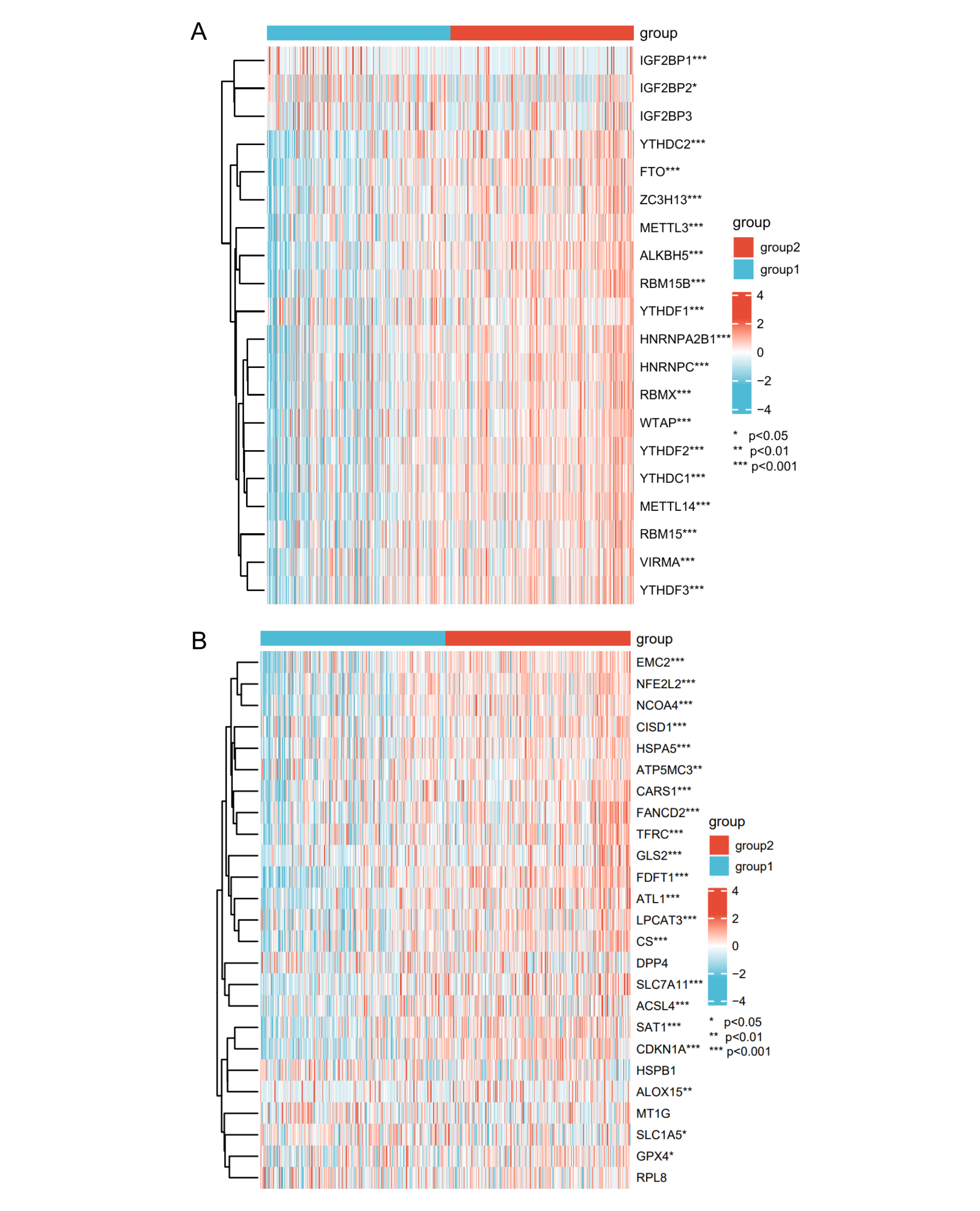

Supplement: Supplementary file 21 [file Image7.TIFF]
