# Supplementary material for: Integrated Bioinformatics Analysis of Serine Racemase as an Independent Prognostic Biomarker in Endometrial Cancer
Source: Front Genet. 2022 Jul 18;13:906291. doi: 10.3389/fgene.2022.906291 (PMC9340001; doi:10.3389/fgene.2022.906291)
Supplement: Supplementary file 3 [file Table1.DOCX]

| Characteristic | Low expression of SRR | High expression of SRR | P value |
| --- | --- | --- | --- |
| n | 276 | 276 |  |
| Age, n (%) |  |  | < 0.001 |
| <=60 | 78 (14.2%) | 128 (23.3%) |  |
| >60 | 198 (36.1%) | 145 (26.4%) |  |
| Clinical stage, n (%) |  |  | 0.067 |
| Stage I | 159 (28.8%) | 183 (33.2%) |  |
| Stage II | 24 (4.3%) | 27 (4.9%) |  |
| Stage III | 74 (13.4%) | 56 (10.1%) |  |
| Stage IV | 19 (3.4%) | 10 (1.8%) |  |
| Primary therapy outcome, n (%) |  |  | 0.276 |
| PD | 14 (2.9%) | 6 (1.2%) |  |
| SD | 3 (0.6%) | 3 (0.6%) |  |
| PR | 6 (1.2%) | 6 (1.2%) |  |
| CR | 212 (44.2%) | 230 (47.9%) |  |
| Race, n (%) |  |  | 0.137 |
| Asian | 6 (1.2%) | 14 (2.8%) |  |
| Black or African American | 51 (10.1%) | 57 (11.2%) |  |
| White | 196 (38.7%) | 183 (36.1%) |  |
| BMI, n (%) |  |  | 0.534 |
| <=30 | 111 (21.4%) | 101 (19.5%) |  |
| >30 | 151 (29.1%) | 156 (30.1%) |  |
| Histological type, n (%) |  |  | < 0.001 |
| Endometrioid | 172 (31.2%) | 238 (43.1%) |  |
| Mixed | 14 (2.5%) | 10 (1.8%) |  |
| Serous | 90 (16.3%) | 28 (5.1%) |  |
| Histologic grade, n (%) |  |  | 0.044 |
| G1 | 44 (8.1%) | 54 (10%) |  |
| G2 | 50 (9.2%) | 70 (12.9%) |  |
| G3 | 174 (32.2%) | 149 (27.5%) |  |
| Residual tumor, n (%) |  |  | 0.014 |
| R0 | 183 (44.3%) | 192 (46.5%) |  |
| R1 | 16 (3.9%) | 6 (1.5%) |  |
| R2 | 12 (2.9%) | 4 (1%) |  |
| Menopause status, n (%) |  |  | 0.002 |
| Pre | 12 (2.4%) | 23 (4.5%) |  |
| Peri | 3 (0.6%) | 14 (2.8%) |  |
| Post | 240 (47.4%) | 214 (42.3%) |  |
| Hormones therapy, n (%) |  |  | 0.689 |
| No | 152 (44.2%) | 145 (42.2%) |  |
| Yes | 22 (6.4%) | 25 (7.3%) |  |
| Diabetes, n (%) |  |  | 0.870 |
| No | 170 (37.7%) | 158 (35%) |  |
| Yes | 62 (13.7%) | 61 (13.5%) |  |
| Radiation therapy, n (%) |  |  | 0.180 |
| No | 149 (28.3%) | 130 (24.7%) |  |
| Yes | 117 (22.2%) | 131 (24.9%) |  |
| Age, median (IQR) | 66 (60, 73) | 62 (55, 70) | < 0.001 |
