# Supplementary material for: Integrated Bioinformatics Analysis of Serine Racemase as an Independent Prognostic Biomarker in Endometrial Cancer
Source: Front Genet. 2022 Jul 18;13:906291. doi: 10.3389/fgene.2022.906291 (PMC9340001; doi:10.3389/fgene.2022.906291)
Supplement: Supplementary file 7 [file Table7.DOCX]

| LncRNAs | GEPIA2.0  OS(HR,P-value) | GEPIA2.0  DFS | StarBase  OS | TCGA TCGA TCGA  OS DSS PFI |
| --- | --- | --- | --- | --- |
| MATN1-AS1 | 1.1(0.82) | 0.79(0.48) | 0.82(0.37) | 0.84(0.409) 0.80(0.387) 0.92(0.647) |
| RAMP2-AS1 | 1.6(0.22) | 0.63(0.17) | 1.65(0.02) | 1.63(0.012) 1.62(0.061) 1.26(0.199) |
| MIR99AHG | 0.92(0.82) | 1.1(0.8) | 1.06(0.78) | 1.23(0.315) 1.49(0.119) 1.19(0.324) |
| SH3BP5-AS1 | 0.87(0.69) | 0.88(0.7) | 0.72(0.13) | 0.77(0.209) 0.80(0.382) 0.85(0.344) |
| MBNL1-AS1 | 1.2(0.68) | 1.1(0.88) | 0.99(0.97) | 1.11(0.609) 1.21(0.453) 1.05(0.772) |
| AC008443.1 | 0.84(0.63) | 0.9(0.76) | 1.02(0.91) | 1.11(0.611) 1.05(0.834) 1.05(0.784) |
| MUC20-OT1 | 2(0.067) | 2.1(0.035) | 1.75(0.0097) | 1.67(0.016) 1.85(0.019) 1.38(0.071) |
| LINC02381 | 1.6(0.22) | 0.93(0.83) | 1.56(0.038) | 1.68(0.014) 2.02(0.008) 1.47(0.031) |
| AL590705.5 | 1.5(0.26) | 1.4(0.33) | 1.36(0.15) | 1.50(0.053) 1.88(0.016) 1.28(0.161) |
| AC68888.1 | 0.69(0.32) | 1.4(0.27) | 0.76(0.21) | 0.95(0.821) 0.90(0.668) 1.07(0.719) |
| AC015712.2 | 0.88(0.71) | 0.79(0.47) | 0.86(0.47) | 1.01(0.944) 1.24(0.397) 1.15(0.434) |
| TSPOAP1-AS1 | 0.49(0.058) | 0.49(0.045) | 0.69(0.081) | 0.67(0.058) 0.48(0.007) 0.74(0.091) |
| ILF3-AS1 | 0.65(0.22) | 0.72(0.33) | 0.97(0.9) | 1.04(0.866) 1.03(0.907) 0.94(0.711) |
| AC012313.1 | 0.99(0.97) | 0.38(0.0082) | 0.83(0.38) | 0.80(0.284) 0.91(0.706) 0.88(0.46) |
| AL137058.2 | 0.77(0.45) | 0.48(0.03) | 0.86(0.49) | 0.82(0.337) 0.82(0.444) 0.75(0.115) |
| AC015871.3 | 0.73(0.36) | 0.7(0.28) | 0.86(0.48) | 0.97(0.9) 1.11(0.668) 1.11(0.559) |
| AL662795.1 | 0.54(0.097) | 0.66(0.21) | 0.85(0.44) | 0.83(0.359) 1.09(0.738) 1.07(0.701) |
| LINC00294 | 1.2(0.68) | 0.6(0.13) | 0.92(0.69) | 1.00(0.996) 0.93(0.761) 0.94(0.722) |
| AC12531.1 | 1.2(0.57) | 1.2(0.51) | 1.64(0.052) | NA NA NA |
