# Supplementary material for: Integrated Bioinformatics Analysis of Serine Racemase as an Independent Prognostic Biomarker in Endometrial Cancer
Source: Front Genet. 2022 Jul 18;13:906291. doi: 10.3389/fgene.2022.906291 (PMC9340001; doi:10.3389/fgene.2022.906291)
Supplement: Supplementary file 10 [file Table2.DOCX]

| Characteristics | Total(N) | Odds Ratio(OR) | P value |
| --- | --- | --- | --- |
| Histologic grade (G3 vs. G1&G2) | 541 | 1.540 (1.091-2.181) | 0.014 |
| Clinical stage (Stage III&Stage IV vs. Stage I&Stage II) | 552 | 1.617 (1.116-2.353) | 0.011 |
| Histological type (Serous vs. Endometrioid) | 528 | 4.448 (2.820-7.202) | <0.001 |
| Primary therapy outcome (PD&SD vs. PR&CR) | 480 | 2.045 (0.912-4.890) | 0.091 |
| Race (White vs. Black or African American) | 487 | 1.197 (0.780-1.840) | 0.410 |
| Age (>60 vs. <=60) | 549 | 2.241 (1.577-3.201) | <0.001 |
| Residual tumor (R1&R2 vs. R0) | 413 | 2.938 (1.431-6.516) | 0.005 |
| Menopause status (Post vs. Pre&Peri) | 506 | 2.766 (1.505-5.328) | 0.001 |

**Abbreviation:**

ACC：adrenocortical carcinoma

BLCA：bladder urothelial carcinoma

BRCA：breast invasive carcinoma

CESC：cervical squamous cell carcinoma and endocervical adenocarcinoma

CHOL：cholangiocarcinoma

COAD：colon carcinoma

DLBC：lymphoid neoplasm diffuse large B-cell lymphoma

ESCA：esophageal carcinoma

GBM： glioblastoma multiforme

HNSC：head and neck squamous cell carcinoma

KICH：kidney chromophobe

KIRC：kidney renal clear cell carcinoma

KIRP：kidney renal papillary cell carcinoma

LAML：acute myeloid leukemia

LGG : brain lower grade glioma

LIHC : liver hepatocellular carcinoma

LUAD : lung adenocarcinoma

LUSC : lung squamous cell carcinoma

MESO : mesothelioma

OV : ovarian serous cystadenocarcinoma

OSCC : oral squamous cell carcinoma

PAAD : pancreatic adenocarcinoma

PCPG : pheochromocytoma and paraganglioma

PRAD：prostate adenocarcinoma

READ：rectum adenocarcinoma

SARC : sarcoma

SKCM : skin cutaneous melanoma

STAD : stomach adenocarcinoma

TGCT : testicular germ cell tumors

THCA : thyroid carcinoma

THYM : thymoma

UCEC : uterine corpus endometrial carcinoma

UCS : uterine carcinosarcoma

UVM : uveal melanoma

EC: endometrial cancer

SRR: serine racemase

ncRNAs: non-coding RNAs

FIGO: Federation of Gynecology and Obstetrics

NMDAR: n-methyl-d-aspartate receptor

UCEC: uterine corpus endometrial carcinoma

TCGA: The Cancer Genome Atlas

GTEx: The Genotype-Tissue Expression

GEO: The Gene Expression Omnibus

HPA: The Human Protein Atlas

CCLE: The Cancer Cell Line Encyclopedia

GSCA: The Gene Set Cancer Analysis

GEPIA2: The Gene Expression Profiling Interactive Analysis

GO: Gene Ontology

KEGG: Kyoto Encyclopedia of Genes and Genomes

GSEA: Gene Set Enrichment Analysis

TIMER: The Tumor Immune Estimation Resource

OS: overall survival

DSS: disease-specific survival

PFI: progress-free interval

DFI: disease-free interval

PFS: progression-free survival

RFS: relapse-free survival

IHC: immunohistochemistry

SNP: [single](#/javascript:;) [nucleotide](#/javascript:;) [polymorphism](#/javascript:;)

SNV: single nucleotide variant

CNV: copy number variation

OR: odds ratio

M6A: N6-methyladenosine

M1A: N1-methyladenosine

M5C: 5-methylcytosine

AUC: area under the curve

HR: hazard ratio

miRNA: microRNA

lncRNA: long non-coding RNA

CeRNA: competing endogenous RNAs

TMB: tumor mutation burden

MSI: [microsatellite](javascript:;) [instability](javascript:;)

MATH: mutant-allele tumor heterogeneity

ICGC: International Cancer Genome Consortium
IC50: half-maximal inhibitory concentration
