# Supplementary material for: Integrated Bioinformatics Analysis of Serine Racemase as an Independent Prognostic Biomarker in Endometrial Cancer
Source: Front Genet. 2022 Jul 18;13:906291. doi: 10.3389/fgene.2022.906291 (PMC9340001; doi:10.3389/fgene.2022.906291)
Supplement: Supplementary file 11 [file Table5.DOCX]

| Characteristics | Total(N) | Univariate analysis | |  | Multivariate analysis | |
| --- | --- | --- | --- | --- | --- | --- |
|  |  | Hazard ratio (95% CI) | P value |  | Hazard ratio (95% CI) | P value |
| Clinical stage | 551 |  |  |  |  |  |
| Stage I&Stage II | 392 | Reference |  |  |  |  |
| Stage III&Stage IV | 159 | 3.169 (2.241-4.481) | **<0.001** |  | 3.049 (2.062-4.509) | **<0.001** |
| Age | 549 |  |  |  |  |  |
| <=60 | 206 | Reference |  |  |  |  |
| >60 | 343 | 1.353 (0.934-1.961) | 0.110 |  |  |  |
| Histological type | 527 |  |  |  |  |  |
| Endometrioid | 409 | Reference |  |  |  |  |
| Serous | 118 | 2.125 (1.466-3.081) | **<0.001** |  | 1.028 (0.639-1.653) | 0.910 |
| Histologic grade | 540 |  |  |  |  |  |
| G1&G2 | 218 | Reference |  |  |  |  |
| G3 | 322 | 2.088 (1.391-3.136) | **<0.001** |  | 1.553 (0.975-2.474) | 0.064 |
| SRR | 551 |  |  |  |  |  |
| High | 275 | Reference |  |  |  |  |
| Low | 276 | 2.295 (1.584-3.326) | **<0.001** |  | 2.053 (1.351-3.121) | **<0.001** |
| Surgical approach | 529 |  |  |  |  |  |
| Minimally Invasive | 208 | Reference |  |  |  |  |
| open | 321 | 0.629 (0.440-0.899) | **0.011** |  | 0.544 (0.373-0.794) | **0.002** |
| Radiation therapy | 527 |  |  |  |  |  |
| Yes | 248 | Reference |  |  |  |  |
| No | 279 | 0.913 (0.643-1.298) | 0.613 |  |  |  |
| Menopause status | 488 |  |  |  |  |  |
| Pre | 35 | Reference |  |  |  |  |
| Post | 453 | 1.552 (0.683-3.527) | 0.294 |  |  |  |
