# Supplementary material for: Integrated Bioinformatics Analysis of Serine Racemase as an Independent Prognostic Biomarker in Endometrial Cancer
Source: Front Genet. 2022 Jul 18;13:906291. doi: 10.3389/fgene.2022.906291 (PMC9340001; doi:10.3389/fgene.2022.906291)
Supplement: Supplementary file 12 [file Table3.DOCX]

| Characteristics | Total(N) | Univariate analysis | |  | Multivariate analysis | |
| --- | --- | --- | --- | --- | --- | --- |
|  |  | Hazard ratio (95% CI) | P value |  | Hazard ratio (95% CI) | P value |
| Clinical stage | 551 |  |  |  |  |  |
| Stage I&Stage II | 392 | Reference |  |  |  |  |
| Stage III&Stage IV | 159 | 3.543 (2.355-5.329) | **<0.001** |  | 3.107 (1.922-5.021) | **<0.001** |
| Age | 549 |  |  |  |  |  |
| <=60 | 206 | Reference |  |  |  |  |
| >60 | 343 | 1.847 (1.160-2.940) | **0.010** |  | 1.873 (1.067-3.286) | **0.029** |
| Histological type | 527 |  |  |  |  |  |
| Endometrioid | 409 | Reference |  |  |  |  |
| Serous | 118 | 2.646 (1.726-4.057) | **<0.001** |  | 0.791 (0.451-1.387) | 0.414 |
| Histologic grade | 540 |  |  |  |  |  |
| G1&G2 | 218 | Reference |  |  |  |  |
| G3 | 322 | 3.281 (1.907-5.643) | **<0.001** |  | 2.695 (1.471-4.939) | **0.001** |
| SRR | 551 |  |  |  |  |  |
| High | 275 | Reference |  |  |  |  |
| Low | 276 | 2.494 (1.595-3.900) | **<0.001** |  | 2.027 (1.208-3.400) | **0.007** |
| Surgical approach | 529 |  |  |  |  |  |
| Minimally Invasive | 208 | Reference |  |  |  |  |
| open | 321 | 0.709 (0.465-1.082) | 0.111 |  |  |  |
| Radiation therapy | 527 |  |  |  |  |  |
| Yes | 248 | Reference |  |  |  |  |
| No | 279 | 1.684 (1.092-2.596) | **0.018** |  | 2.218 (1.384-3.555) | **<0.001** |
| Menopause status | 488 |  |  |  |  |  |
| Pre | 35 | Reference |  |  |  |  |
| Post | 453 | 0.850 (0.392-1.843) | 0.681 |  |  |  |
