# Supplementary material for: Integrated Bioinformatics Analysis of Serine Racemase as an Independent Prognostic Biomarker in Endometrial Cancer
Source: Front Genet. 2022 Jul 18;13:906291. doi: 10.3389/fgene.2022.906291 (PMC9340001; doi:10.3389/fgene.2022.906291)
Supplement: Supplementary file 15 [file Table4.DOCX]

| Characteristics | Total(N) | Univariate analysis | |  | Multivariate analysis | |
| --- | --- | --- | --- | --- | --- | --- |
|  |  | Hazard ratio (95% CI) | P value |  | Hazard ratio (95% CI) | P value |
| Clinical stage | 549 |  |  |  |  |  |
| Stage I&Stage II | 390 | Reference |  |  |  |  |
| Stage III&Stage IV | 159 | 7.030 (4.093-12.077) | **<0.001** |  | 5.450 (2.908-10.214) | **<0.001** |
| Age | 547 |  |  |  |  |  |
| <=60 | 206 | Reference |  |  |  |  |
| >60 | 341 | 1.215 (0.724-2.042) | 0.461 |  |  |  |
| Histological type | 525 |  |  |  |  |  |
| Endometrioid | 407 | Reference |  |  |  |  |
| Serous | 118 | 3.452 (2.047-5.824) | **<0.001** |  | 0.755 (0.390-1.461) | 0.403 |
| Histologic grade | 538 |  |  |  |  |  |
| G1&G2 | 218 | Reference |  |  |  |  |
| G3 | 320 | 7.851 (3.137-19.651) | **<0.001** |  | 6.415 (2.405-17.111) | **<0.001** |
| SRR | 549 |  |  |  |  |  |
| High | 274 | Reference |  |  |  |  |
| Low | 275 | 3.164 (1.772-5.650) | **<0.001** |  | 3.192 (1.579-6.454) | **0.001** |
| Surgical approach | 527 |  |  |  |  |  |
| Minimally Invasive | 208 | Reference |  |  |  |  |
| open | 319 | 0.661 (0.396-1.103) | 0.113 |  |  |  |
| Radiation therapy | 525 |  |  |  |  |  |
| Yes | 248 | Reference |  |  |  |  |
| No | 277 | 1.670 (0.979-2.849) | 0.060 |  | 2.276 (1.263-4.103) | **0.006** |
| Menopause status | 486 |  |  |  |  |  |
| Pre | 35 | Reference |  |  |  |  |
| Post | 451 | 0.858 (0.343-2.142) | 0.743 |  |  |  |
