# Supplementary material for: Integrated Bioinformatics Analysis of Serine Racemase as an Independent Prognostic Biomarker in Endometrial Cancer
Source: Front Genet. 2022 Jul 18;13:906291. doi: 10.3389/fgene.2022.906291 (PMC9340001; doi:10.3389/fgene.2022.906291)
Supplement: Supplementary file 17 [file Table6.DOCX]

| LncRNAs | GEPIA2.0  OS(HR,P-value) | GEPIA2.0  DFS | StarBase  OS | TCGA TCGA TCGA  OS DSS PFI |
| --- | --- | --- | --- | --- |
| AC008969.1 | 1.2(0.57) | 0.71(0.3) | 1.88(0.44) | 1.26(0.268) 1.61(0.065) 1.21(0.286) |
| LINC00963 | 0.69(0.29) | 0.65(0.19) | 0.83(0.37) | 0.76(0.181) 0.76(0.288) 0.70(0.048) |
| C1RL-AS1 | 1.1(0.87) | 0.73(0.34) | 0.98(0.91) | 1.24(0.294) 1.33(0.261) 1.10(0.59) |
| XIST | 1.4(0.37) | 1.2(0.63) | 0.92(0.69) | 0.96(0.857) 0.94(0.799) 0.88(0.467) |
| SNHG7 | 0.5(0.06) | 1.3(0.44) | 0.76(0.19) | 0.87(0.496) 0.78(0.322) 0.82(0.252) |
| AC008443.1 | 0.84(0.63) | 0.9(0.76) | 1.02(0.91) | 0.11(0.611) 1.05(0.834) 1.05(0.784) |
| LINC01278 | 1.3(0.42) | 0.98(0.94) | 0.78(0.23) | 0.91(0.633) 1.09(0.722) 1.02(0.929) |
| TTN-AS1 | 0.87(0.7) | 0.86(0.64) | 0.91(0.65) | 1.05(0.805) 1.09(0.743) 0.90(0.56) |
| SLC25A21-AS1 | 0.75(0.43) | 0.61(0.14) | 0.92(0.68) | 1.2(0.373) 1.14(0.61) 1.08(0.647) |
| AC24075.2 | 0.77(0.46) | 0.65(0.2) | 0.68(0.067) | 0.72(0.118) 0.65(0.09) 0.66(0.021) |
| HEIH | 0.84(0.63) | 0.91(0.78) | 0.96(0.83) | 0.9(0.614) 0.72(0.197) 0.98(0.931) |
| AL662795.1 | 0.54(0.097) | 0.66(0.21) | 0.85(0.44) | 0.83(0.359) 1.09(0.738) 1.07(0.701) |
| LINC00294 | 1.2(0.68) | 0.6(0.13) | 0.92(0.69) | 1.0(0.996) 0.93(0.761) 0.94(0.722) |
